# Association of Inflammation-Related Gene Polymorphisms With Susceptibility and Radiotherapy Sensitivity in Head and Neck Squamous Cell Carcinoma Patients in Northeast China

**DOI:** 10.3389/fonc.2021.651632

**Published:** 2021-06-04

**Authors:** Ying Li, Li Zhu, Hongmin Yao, Ye Zhang, Xiangyu Kong, Liping Chen, Yingqiu Song, Anna Mu, Xia Li

**Affiliations:** Department of Radiation Oncology, Cancer Hospital of China Medical University, Liaoning Cancer Hospital & Institute, and Key Laboratory of Tumor Radiosensitization and Normal Tissue Radioprotection of Liaoning Province, Shenyang, China

**Keywords:** inflammation-related gene, SNP, HNSCC, risk, radiotherapy sensitivity

## Abstract

**Background:**

Inflammation-related gene polymorphisms are some of the most important determinants for cancer susceptibility, clinical phenotype diversity, and the response to radiotherapy and chemotherapy. However, the relationship between these polymorphisms and head and neck squamous cell carcinoma (HNSCC) remains unclear. The aim of this study was to investigate the role of inflammation-related gene polymorphisms in the developmental risk and radiotherapy sensitivity of HNSCC.

**Methods:**

The Matrix-Assisted Laser Desorption Ionization Time of Flight (MALDI-TOF) genotyping system was used to genotype 612 individuals from a Chinese population for 28 inflammation-related gene polymorphisms.

**Results:**

The protein kinase B (AKT1) rs1130233 TT, dominance model (CT+TT vs. CC), recessive model (TT vs. CT+CC), and rs2494732 CC genotypes were associated with reduced risk of HNSCC (*P*=0.014; *P*=0.041; *P*=0.043). The polymeric immunoglobulin receptor (PIGR) rs291097 GA, dominance model (GA+AA vs. GG), and rs291102 dominance model (GA+AA vs. GG) were associated with increased risk of HNSCC (*P*=0.025; *P*=0.025; *P*=0.040). The interleukin-4 receptor-α (IL-4RA) rs1801275 AA genotype was significantly correlated with increased radiotherapy sensitivity of HNSCC patients (*P*=0.030). In addition, age ≤ 60 years, non-smoker status, and normal levels of squamous cell carcinoma antigen (SCC) were found to be associated with increased radiotherapy sensitivity of HNSCC patients (*P*=0.033; *P*=0.033; *P*=0.030).

**Conclusion:**

The AKT1 rs1130233, AKT1 rs2494732, PIGR rs291097, and PIGR rs291102 polymorphisms were significantly related to the risk of HNSCC. The IL-4RA rs1801275 polymorphism, age ≤ 60 years, non-smoker status, and normal levels of SCC were significantly associated with increased radiotherapy sensitivity of HNSCC.

## Introduction

Head and neck squamous cell carcinoma (HNSCC) is a general term for a set of different tumors located in the lips, oral cavity, pharynx (nasopharynx, oropharynx and hypopharynx), as well as the larynx, salivary glands, and thyroid glands (1). HNSCC is sixth in the world in overall incidence, and is also a major cancer type that leads to death (1). The initiation and development of HNSCC is a multistep process influenced by various genetic and environmental factors. Tobacco and alcohol consumption are the most classical risk factors associated with its development. At least 75% of HNSCC cases are attributable to the combination of both tobacco and alcohol use (2). However, the role of genetic factors in head and neck squamous cell carcinogenesis is largely unknown.

Single nucleotide polymorphisms (SNPs) are a class of genetic factors that have been implicated in HNSCC susceptibility and determine inter-individual variations in HNSCC risk. Genetic polymorphisms can weaken intrinsic protective mechanisms and increase the damage caused by environmental carcinogens (3). Carriers of susceptible genotypes are at a greater risk of developing cancer than those with resistant genotypes under similar conditions (3). Therefore, genetic factors may play a crucial role in HNSCC risk and clinical outcome.

Inflammation is an important cellular process that can be activated in response to tissue damage, infections, and other cellular stress factors^6^. There is a relationship between inflammation and the development of many cancers where tumorigenesis was initiated at the site of inflammation (4, 5). Interleukin-1 (IL-1) is a pleiotropic cytokine involved in the initiation of immune and inflammatory responses. The IL-1 gene family has been reported to play a crucial role in the pathogenesis of various cancers (6–9). The interleukin-1 receptor antagonist (IL-1RN) polymorphism is associated with cervical cancer (10). Additionally, there is a pro-inflammatory cytokine haplotype (IL-6 CC, IL-10 GG, TNF-α AA) that is associated with adverse prognosis that may act through an inflammatory-mediated mechanism (11). Furthermore, protein kinase B (AKT1) is an important downstream effector of the gene of phosphate and tension homology deleted on chromosome ten/phosphoinositide 3-kinase/protein kinase B (PTEN/PI3K/AKT) signal transduction pathway. Aberrant expression and genetic variation of the *AKT1* gene are suggested to be involved in several types of human cancers, including oral squamous cell carcinoma (OSCC) (12). The AKT1 rs1130214 and rs3803300 polymorphisms were related to OSCC susceptibility in a Chinese Han population (12). The polymeric immunoglobulin receptor (PIGR) 1739C>T is a missense mutation that results in an alanine residue being changed to valine near an endoproteolytic cleavage site. This variant can alter the efficiency of PIGR to release the Epstein–Barr virus immunoglobulin A (IgA-EBV) complex and consequently increase the susceptibility of populations in endemic areas to develop NPC (13). PIGR 8880C>T is also related to NPC susceptibility (14). Additionally, the cyclooxygenase-2 (COX-2) gene (*PTGS2*) rs5275 variant contributes to NPC risk in a Chinese population (15).

Chronic inflammation promotes genetic and epigenetic aberrations that result in various pathogeneses. These changes may be useful biomarkers in liquid biopsies for early detection and prevention of various cancers (16). To achieve our aim, analysis of candidate genes in a Chinese population was performed to study 28 SNPs in inflammation-related genes that could possibly be associated with the risk of developing HNSCC.

## Materials and Methods

### Research Design and Study Population

The study design was approved by the Human Ethics Committee of Liaoning Cancer Hospital (Shenyang, China). Each individual provided written informed consent during an epidemiological investigation. Patients were from Liaoning Cancer Hospital and received surgical resection or needle biopsy diagnosis/treatment between 2018 and 2019. The control participants were recruited from health check center in Liaoning Province hospital between 2018 and 2019. The HNSCC patient group and the control group were matched at a 1:2 ratio. All diagnoses of HNSCC patients were based on histopathological examinations. Information regarding smoking habits, alcohol consumption, and family history in cases were acquired by a “face-to-face” questionnaire survey. We collected fasting venous blood from each one and stored the samples at −20°C as serum and clotted cells.

To further evaluate the relationship of polymorphisms with clinicopathological parameters of HNSCC, histology or clinical data were assessed according to World Health Organization criteria. Additionally, tumor-node-metastasis (TNM) staging was performed according to the 8th edition of the International Union Against Cancer (UICC)/American Joint Committee on Cancer (AJCC) (2017) criteria (17).

### SNP Selection

A compilation of genes involved in the inflammatory response was conducted on the basis of a published panel of inflammation-associated genes (6, 9, 13–15, 18–44) and the NCBI-Gene website analysis (https://www.ncbi.nlm.nih.gov/gene/). In this study, we selected 16 genes and 28 SNPs for analysis. They are as follows: AKT1 rs130233 and rs2494732; complement C3d receptor 2 (CR2) rs3813946; IL10 rs1800871, rs1800872, and rs1800896; IL1A rs17561; IL1B rs1143627, rs16944, and rs1143634; IL1RN rs419598; IL21R rs2189521; IL4 rs2243250 and rs2227284; IL4RA rs1801275; IL6 rs1800796; PIGR rs291097 and rs291102; tumor necrosis factor (TNF) rs1799964, rs1800629, rs361525, rs1800630 and rs1799724; TNFRSF1A rs4149570; TNFSF7 rs7259857; COX-2 rs5275 and rs20417; B-cell lymphoma-2 (BCL2) rs2279115.

### SNP Genotyping

Genomic DNA was extracted from peripheral blood samples obtained from the study participants using the phenol-cholesterol method according to a standard procedure (45). The Matrix-Assisted Laser Desorption Ionization Time of Flight (MALDI-TOF) genotyping system was used to genotype 612 individuals for 28 inflammation-related gene polymorphisms. MALDI-TOF is a medium-to-high-throughput technology platform that takes both sensitivity and specific into account and used mass spectrometry for direct detection (46). Amplification and extension primers were designed by BGI. The charged analytes were detected and measured using time of flight analyzers. During MALDI-TOF analysis, the m/z ratio of an ion was measured by determining the time required for the ion to travel the length of the flight tube (47, 48). Primers sequences are listed in **Supplementary Table 1**.

### Radiosensitivity Analysis

Radiosensitivity analysis was done according to the new response evaluation criteria for solid tumors: Revised response evaluation criteria in solid tumors (RECIST) guideline (version 1.1) (49). Patients who were sensitive to radiation therapy were categorized as either complete response (CR) or partial response (PR). Patients who were not sensitive to radiation therapy were categorized as either progressive disease (PD) or stable disease (SD). Radiosensitivity was assessed one month after radiotherapy, and the results were compared with the MRI image before radiotherapy. The criteria for classification are as follows:

CR: patients had a disappearance of all target lesions and any pathological lymph nodes (whether target or non-target) were required to have a short axis reduction to <10 mm.
PR: patients were required to have at least a 30% decrease in the sum of the diameters of target lesions, using the baseline sum diameters as a reference.
PD: patients were required to have at least a 20% increase in the sum of the diameters of target lesions, using the smallest sum of the study as a reference. In addition to the relative increase of 20%, the sum was also required to demonstrate an absolute increase of at least 5 mm. Patients that had an appearance of one or more new lesions were also categorized as PD.
SD: patients were required to have neither a sufficient level of shrinkage to qualify for PR nor a sufficient amount of increase to qualify for PD. The smallest sum diameters were used as references.

### Statistical Analysis

Statistical analysis was performed using SPSS (version 22.0). Adjusted odds ratios (ORs) and 95% confidence intervals (CIs) for the relationships between both SNPs and disease risk were calculated by multivariable logistic regression, with adjustments for gender and age. If stratified by sex, then the age was adjusted; if stratified by age, then the sex was adjusted. Chi-squared tests were used to assess the correlation between different genotypes and the clinicopathological parameters and radiosensitivity of HNSCC patients.

## Results

### Baseline Patient Characteristics

To analyze the risk of HNSCC, the study subjects included 211 patients with HNSCC and 401 age- and sex-matched control subjects. The comparisons of baseline characteristics between cases and controls are shown in **Table 1**. There was a significant difference in both age and sex distribution between the HNSCC group and the control group. The overall mean age and mean age of menarche differed significantly between cases and controls (both *P*<0.001). In cases, the mean menopausal age was 58.00 years and only a small proportion of cases had a family history of cancer (15.2%). In cases with invasion depth, 55.2% and 44.8% of cases were in T1-2 and T3-4, respectively. Tumor stages I-II (23.7%) and III-IV (76.3%) accounted for the majority of HNSCC cases, whereas 69.6% of cases had positive lymph nodes and 5.9% of cases had metastasis (**Table 1**).

**Table 1:** The baseline characteristics of the objects.

Characteristics		Cases	Controls	P value
Sample size		211	401	
Age				**<0.001**
	Mean±SD	56.83±0.75	36.25±0.63	
	Mmenarche	58	32	
	Range	14-90	17-73	
Gender	Female	49(23.2%)	175(43.6%)	**<0.001**
	Male	162(76.8%)	226(56.4%)	
T stage	1-2	96(55.2%)		
	3-4	78(44.8%)		
N stage	Negative	55(30.4%)		
	Positive	126(69.6%)		
M stage	Negative	177(94.1%)		
	Positive	11(5.9%)		
Clinical stage	I-II	44(23.7%)		
	III-IV	142(76.3%)		
Smoking	No	102(48.3%)		
	Yes	109(51.7%)		
Drinking	No	106(50.2%)		
	Yes	105(49.8%)		
Family history of cancer	No	179(84.8%)		
	Yes	32(15.2%)		
SCC	Normal	80(79.2%)		
	Increased	21(20.8%)		
CEA	Normal	60(93.8%)		
	Increased	4(6.3%)		
CYFRA	Normal	16(48.5%)		
	Increased	17(51.5%)		
EBV	Negative	30(83.3%)		
	Positive	6(16.7%)		
Blood type	A	40(33.6%)		
	B	32(26.9%)		
	AB	14(11.8%)		
	O	33(27.7%)		

There was a significant difference in both age and sex distribution between the HNSCC group and the control group (both P<0.001). The case group is significantly older than the control group. Men are significantly more than women, especially in the case group.

### Association of 28 Inflammation-Associated Gene SNPs With HNSCC Risk

Multivariable logistic regression was used to investigate the association of 28 inflammation-associated gene SNPs with HNSCC risk. The results indicated that the AKT1 rs1130233 and rs2494732 SNPs, as well as the PIGR rs291097 and rs291102 SNPs, had a significant association with HNSCC risk progression (**Table 2**). We also found that the carriers of the AKT1 rs1130233 TT genotype, dominance model (CT+TT vs. CC), recessive model (TT vs. CT+CC), or the AKT1 rs2494732 CC genotype had reduced risk of HNSCC (*P*<0.05), whereas those with the PIGR rs291097 GA genotype, dominance model (GA+ AA vs. GG), or PIGR rs291102 dominance model (GA+ AA vs. GG) had an increased risk of HNSCC (*P*<0.05). However, we found no significant differences with the other 24 SNPs in HNSCC risk progression (**Table 2**).

**Table 2:** Association of 28 inflammation-associated gene SNPs with HNSCC risk.

Genetype	SNP	Cases	Controls	P value	P value	OR (95%CI)
AKT1	rs1130233	N=208	N=400	**0.020**		
	CC	58(27.9%)	77(19.3%)		/	1(Ref)
	CT	98(47.1%)	189(47.3%)		0.149	0.65(0.36,1.17)
	TT	52(25.0%)	134(33.5%)		**0.014**	0.45(0.24,0.85)
	CT+TT vs. CC	/	/		**0.041**	0.57(0.33,0.98)
	TT vs.CT+CC	/	/		**0.046**	0.60(0.36,0.99)
AKT1	rs2494732	N=209	N=395	0.678		
	TT	18(8.6%)	27(6.8%)		/	1(Ref)
	CT	97(46.4%)	158(40.0%)		0.220	0.56(0.22,1.41)
	CC	94(45.0%)	210(53.2%)		**0.043**	0.38(0.15,0.97)
	CT+CC vs. TT	/	/		0.089	0.46(0.19,1.13)
	CC vs.CT+TT	/	/		0.073	0.66(0.42,1.04)
CR2	rs3813946	N=209	N=396	0.309		
	TT	154(73.7%)	313(79.0%)		/	1(Ref)
	CT	53(25.4%)	79(19.9%)		0.825	0.94(0.55,1.62)
	CC	2(1.0%)	4(1.0%)		0.166	0.24(0.03,1.81)
	CT+CC vs. TT	/	/		0.612	0.87(0.51,1.49)
	CC vs.CT+TT	/	/		0.148	0.22(0.03,1.70)
IL10	rs1800871	N=208	N=400	0.861		
	AA	90(43.3%)	164(41.0%)		/	1(Ref)
	GA	98(47.1%)	197(49.3%)		0.395	0.82(0.51,1.31)
	GG	20(9.6%)	39(9.8%)		0.572	1.27(0.55,2.91)
	GA+GG vs. AA	/	/		0.535	0.86(0.55,1.37)
	GG vs. GA+AA	/	/		0.390	1.40(0.65,3.04)
IL10	rs1800872	N=208	N=400	0.861		
	TT	90(43.3%)	164(41.0%)		/	1(Ref)
	GT	98(47.1%)	197(49.3%)		0.395	0.82(0.51,1.31)
	GG	20(9.6%)	39(9.8%)		0.572	1.27(0.55,2.91)
	GT+GG vs.TT	/	/		0.535	0.86(0.55,1.37)
	GG vs.GT+TT	/	/		0.390	1.40(0.65,3.04)
IL10	rs1800896	N=209	N=400	0.297		
	TT	174(83.3%)	322(80.5%)		/	1(Ref)
	CT	33(15.8%)	77(19.3%)		0.552	0.84(0.46,1.51)
	CC	2(1.0%)	1(0.3%)		0.656	1.89(0.12,30.68)
	CT+CC vs. TT	/	/		0.610	0.86(0.48,1.54)
	CC vs.CT+TT	/	/		0.648	1.90(0.12,30.24)
IL1A	rs17561	N=208	N=400	0.833		
	CC	166(79.8%)	327(81.8%)		/	1(Ref)
	CA	40(19.2%)	69(17.3%)		0.754	1.10(0.60,2.01)
	AA	2(1.0%)	4(1.0%)		0.869	1.21(0.13,11.72)
	CA+AA vs. CC	/	/		0.738	1.11(0.61,1.99)
	AA vs.CA+CC	/	/		0.882	1.19(0.12,11.72)
IL1B	rs1143627	N=208	N=394	0.588		
	AA	51(24.5%)	111(28.2%)		/	1(Ref)
	AG	107(51.4%)	188(47.7%)		0.949	0.98(0.58,1.67)
	GG	50(24.0%)	95(24.1%)		0.403	0.76(0.40,1.45)
	AG+GG vs. AA	/	/		0.649	0.90(0.54,1.51)
	GG vs. AG+AA	/	/		0.388	0.79(0.46,1.35)
IL1B	rs16944	N=209	N=397	0.710		
	GG	52(24.9%)	111(28.0%)		/	1(Ref)
	GA	106(50.7%)	191(48.1%)		0.881	0.96(0.56,1.63)
	AA	51(24.4%)	95(23.9%)		0.469	0.79(0.42,1.50)
	GA+AA vs. GG	/	/		0.686	0.90(0.54,1.51)
	AA vs.GA+GG	/	/		0.493	0.83(0.48,1.42)
IL1B	rs1143634	N=209	N=400	0.761		
	GG	199(95.2%)	381(95.3%)		/	1(Ref)
	GA	10(4.8%)	18(4.5%)		0.861	1.10(0.38,3.17)
	AA	0(0.0%)	1(0.3%)		NA	5.06×10^-7^(5.06×10^-7^,5.06×10^-7^)
	GA+AA vs. GG	/	/		0.864	1.10(0.38,3.16)
	AA vs.GA+GG	/	/		NA	4.59×10^-7^(4.59×10^-7^,4.59×10^-7^)
IL1RN	rs419598	N=143	N=393	0.292		
	TT	128(89.5%)	336(85.5%)		/	1(Ref)
	CT	13(9.1%)	54(13.7%)		0.122	0.52(0.22,1.19)
	CC	2(1.4%)	3(0.8%)		0.713	1.49(0.18,12.33)
	CT+CC vs. TT	/	/		0.178	0.58(0.26,1.28)
	CC vs.CT+TT	/	/		0.666	1.57(0.20,12.39)
IL21R	rs2189521	N=208	N=395	0.050		
	TT	131(63.0%)	208(52.7%)		/	1(Ref)
	CT	67(32.2%)	160(40.5%)		0.280	0.77(0.47,1.24)
	CC	10(4.8%)	27(6.8%)		0.613	0.78(0.30,2.05)
	CT+CC vs. TT	/	/		0.267	0.77(0.48,1.22)
	CC vs.CT+TT	/	/		0.778	0.87(0.32,2.34)
IL4	rs2243250	N=209	N=395	0.427		
	CC	9(4.3%)	13(3.3%)		/	1(Ref)
	CT	76(36.4%)	127(32.2%)		0.652	0.76(0.23,2.54)
	TT	124(59.3%)	255(64.6%)		0.384	0.55(0.14,2.12)
	CT+TT vs. CC	/	/		0.468	0.63(0.18,2.20)
	TT vs.CT+CC	/	/		0.251	0.76(0.48,1.21)
IL4	rs2227284	N=209	N=395	0.344		
	TT	144(68.9%)	294(74.4%)		/	1(Ref)
	GT	60(28.7%)	94(23.8%)		0.409	1.24(0.74,2.09)
	GG	5(2.4%)	7(1.8%)		0.336	2.54(0.38,16.88)
	GT+GG vs.TT	/	/		0.317	1.30(0.78,2.16)
	GG vs.GT+TT	/	/		0.370	2.24(0.38,13.07)
IL4RA	rs1801275	N=207	N=400	0.116		
	AA	152(73.4%)	272(68.0%)		/	1(Ref)
	GA	53(25.6%)	114(28.5%)		0.995	1.00(0.60,1.67)
	GG	2(1.0%)	14(3.5%)		0.200	0.31(0.05,1.85)
	GA+GG vs. AA	/	/		0.756	0.92(0.56,1.52)
	GG vs. GA+AA	/	/		0.200	0.31(0.05,1.87)
IL6	rs1800796	N=209	N=395	0.942		
	GG	26(12.4%)	47(11.9%)		/	1(Ref)
	CG	87(41.6%)	170(43.0%)		0.852	1.08(0.49,2.38)
	CC	96(45.9%)	178(45.1%)		0.487	1.32(0.61,2.84)
	CG+CC vs.GG	/	/		0.646	1.19(0.57,2.50)
	CC vs.CG+GG	/	/		0.386	1.23(0.77,1.94)
PIGR	rs291097	N=209	N=400	0.125		
	GG	188(90.0%)	372(93.0%)		/	1(Ref)
	GA	21(10.0%)	28(7.0%)		**0.025**	2.49(1.12,5.53)
	AA	0(0%)	0(0.0%)		NA	NA
	GA+AA vs. GG	/	/		**0.025**	2.49(1.12,5.53)
	AA vs.GA+GG	/	/		NA	NA
PIGR	rs291102	N=208	N=396	0.794		
	GG	165(79.3%)	323(81.6%)		/	1(Ref)
	GA	41(19.7%)	70(17.7%)		0.054	1.82(0.99,3.35)
	AA	2(1.0%)	3(0.8%)		0.291	3.76(0.32,43.88)
	GA+AA vs. GG	/	/		**0.040**	1.86(1.03,3.38)
	AA vs.GA+GG	/	/		0.349	3.17(0.28,35.45)
TNF	rs1799964	N=209	N=395	0.732		
	TT	124(59.3%)	246(62.3%)		/	1(Ref)
	CT	74(35.4%)	132(33.4%)		0.388	1.24(0.76,2.01)
	CC	11(5.3%)	17(4.3%)		0.280	2.03(0.56,7.29)
	CT+CC vs. TT	/	/		0.290	1.29(0.81,2.05)
	CC vs.CT+TT	/	/		0.346	1.79(0.53,5.99)
TNF	rs1800629	N=209	N=396	0.725		
	GG	0(0%)	347(87.6%)		/	1(Ref)
	GA	208(99.5%)	47(11.9%)		NA	NA
	AA	1(0.5%)	2(0.5%)		NA	NA
	GA+AA vs. GG	/	/		NA	NA
	AA vs.GA+GG	/	/		0.470	0.36(0.02,5.74)
TNFRSF1A	rs4149570	N=205	N=395	0.370		
	CC	43(21.0%)	101(25.6%)		/	1(Ref)
	CA	102(49.8%)	194(49.1%)		0.439	1.27(0.69,2.34)
	AA	60(29.3%)	100(25.3%)		0.305	1.39(0.74,2.61)
	CA+AA vs. CC	/	/		0.326	1.33(0.75,2.34)
	AA vs.CA+CC	/	/		0.451	1.22(0.73,2.03)
TNFSF7	rs7259857	N=209	N=396	0.804		
	TT	166(79.4%)	322(81.3%)		/	1(Ref)
	CT	40(19.1%)	70(17.7%)		0.998	1.00(0.54,1.85)
	CC	3(1.4%)	4(1.0%)		0.241	2.86(0.49,16.59)
	CT+CC vs. TT	/	/		0.757	1.10(0.61,1.98)
	CC vs.CT+TT	/	/		0.239	2.87(0.50,16.60)
TNF	rs361525	N=209	N=396	0.467		
	GG	191(91.4%)	364(91.9%)		/	1(Ref)
	GA	18(8.6%)	32(8.1%)		0.640	1.21(0.54,2.73)
	AA	0(0%)	0(0%)		NA	NA
	GA+AA vs. GG	/	/		0.640	1.21(0.54,2.73)
	AA vs.GA+GG	/	/		NA	NA
TNF	rs1800630	N=207	N=395	0.899		
	CC	141(68.1%)	274(69.4%)		/	1(Ref)
	CA	59(28.5%)	110(27.8%)		0.740	1.09(0.65,1.82)
	AA	7(3.4%)	11(2.8%)		0.277	2.30(0.51,10.35)
	CA+AA vs. CC	/	/		0.591	1.15(0.70,1.88)
	AA vs.CA+CC	/	/		0.327	2.06(0.49,8.75)
TNF	rs1799724	N=205	N=398	0.893		
	CC	153(74.6%)	302(75.9%)		/	1(Ref)
	CT	48(23.4%)	90(22.6%)		0.984	1.01(0.59,1.73)
	TT	4(2.0%)	6(1.5%)		0.500	2.17(0.23,20.75)
	CT+TT vs. CC	/	/		0.888	1.04(0.61,1.77)
	TT vs.CT+CC	/	/		0.495	2.22(0.23,21.93)
COX-2	rs5275	N=209	N=396	0.848		
	AA	139(66.5%)	270(68.2%)		/	1(Ref)
	GA	65(31.1%)	115(29.0%)		0.755	1.08(0.66,1.78)
	GG	5(2.4%)	11(2.8%)		0.945	0.94(0.16,5.48)
	GA+GG vs. AA	/	/		0.775	1.07(0.66,1.75)
	GG vs. GA+AA	/	/		0.927	0.92(0.16,5.22)
COX-2	rs20417	N=208	N=393	0.881		
	CC	188(90.4%)	358(91.1%)		/	1(Ref)
	CG	19(9.1%)	34(8.7%)		0.755	0.87(0.37,2.05)
	GG	1(0.5%)	1(0.3%)		0.867	2.34(0.00,47610.96)
	CG+GG vs.CC	/	/		0.767	0.88(0.38,2.06)
	GG vs.CG+CC	/	/		0.860	2.30(0.00,28090.30)
BCL2	rs2279115	N=209	N=395	0.470		
	GG	96(45.9%)	166(42.0%)		/	1(Ref)
	GT	88(42.1%)	169(42.8%)		0.944	1.02(0.63,1.64)
	TT	25(12.0%)	60(15.2%)		0.218	0.61(0.28,1.34)
	GT+TT vs.GG	/	/		0.728	0.92(0.58,1.46)
	TT vs.GT+GG	/	/		0.210	0.64(0.32,1.28)

In the case group and the control group, there were significantly more people carrying the AKT1 rs1130233 heterozygous CT genotype than those carrying the wild type and the mutant type(P=0.020). The carriers of the AKT1 rs1130233 TT genotype, dominance model (CT+TT vs. CC), recessive model (TT vs. CT+CC), or the AKT1 rs2494732 CC genotype had reduced risk of HNSCC (P=0.014, P=0.041, P=0.046, P=0.043), whereas those with the PIGR rs291097 GA genotype, dominance model (GA+ AA vs. GG), or PIGR rs291102 dominance model (GA+ AA vs. GG) had an increased risk of HNSCC (P=0.025, P=0.025, P=0.040).

### Stratified Analysis of the Association of 28 Inflammation-Associated Gene SNPs With HNSCC Risk

In stratified analyses, we found that the IL-1RN rs419598 TT genotype and dominance model (CT+TT vs. CC) conferred a 0.12-fold and 0.16-fold reduction in HNSCC progression, respectively, in individuals older than age 60. However, in those age 60 or younger, the AKT1 rs1130233 TT genotype and dominance model (CT+TT vs. CC), IL-21R rs2189521 CT genotype and dominance model (CT+ CC vs. TT), and BCL2 rs2279115 recessive model (TT vs. GT+GG) conferred a 0.48-fold, 0.57-fold, 0.61-fold, 0.60-fold, and 0.49-fold reduction in HNSCC progression, respectively. In addition, in men, the AKT1 rs1130233 TT genotype and dominance model (CT+TT vs. CC) and the BCL2 rs2279115 TT genotype and recessive model (TT vs. GT+GG) conferred a 0.37-fold, 0.43-fold, 0.37-fold, and 0.41-fold reduction in HNSCC progression, respectively. In women, the IL-21R rs2189521 CT genotype and dominance model (CT+TT vs. TT) conferred a 0.39-fold and 0.43-fold reduction in HNSCC progression, respectively. However, the PIGR rs291097 GA genotype and dominance model (GA+AA vs. GG) and the TNF rs1800630 AA genotype conferred a 3.43-fold, 3.43-fold, and 9.42-fold increase in HNSCC progression, respectively. All these stratified analysis results are shown in **Table 3**.

**Table 3:** Stratified analysis of the association of 28 inflammation-associated gene SNPs with HNSCC risk.

Genetype	SNP	Cases	Controls	P value	P value	OR (95%CI)
Age>60						
AKT1	rs1130233	N=84	N=17	0.332		
	CC	21(25.0%)	2(11.8%)		/	1(Ref)
	CT	41(48.8%)	8(47.1%)		0.610	0.64(0.124,3.52)
	TT	22(26.2%)	7(41.2%)		0.150	0.29(0.05,1.57)
	CT+TT vs. CC	/	/		0.302	0.44(0.09,2.10)
	TT vs.CT+CC	/	/		0.165	0.45(0.15,1.38)
AKT1	rs2494732	N=85	N=17	0.460		
	TT	7(8.2%)	0(0%)		/	1(Ref)
	CT	39(45.9%)	9(52.9%)		NA	3.55×10^-8^(3.55×10^-8^,3.55×10^-8^)
	CC	39(45.9%)	8(47.1%)		NA	2.74×10^-8^(2.74×10^-8^,2.74×10^-8)^
	CT+CC vs. TT	/	/		NA	8.80×10^-8^(8.80×10^-8^,8.80×10^-8)^
	CC vs.CT+TT	/	/		0.851	0.90(0.31,2.60)
CR2	rs3813946	N=85	N=17	0.684		
	TT	62(72.9%)	14(82.4%)		/	1(Ref)
	CT	22(25.9%)	3(17.6%)		0.442	1.70(0.44,6.57)
	CC	1(1.2%)	0(0%)		NA	NA
	CT+CC vs. TT	/	/		0.411	1.76(0.46,6.79)
	CC vs.CT+TT	/	/		NA	NA
IL10	rs1800871	N=83	N=17	0.186		
	AA	37(44.6%)	5(29.4%)		/	1(Ref)
	GA	40(48.2%)	12(70.6%)		0.176	0.45(1.14,1.43)
	GG	6(7.2%)	0(0%)		NA	NA
	GA+GG vs. AA	/	/		0.258	0.52(0.17,1.62)
	GG vs. GA+AA	0.1	/		NA	NA
IL10	rs1800872	N=83	N=17	0.186		
	TT	37(44.6%)	5(29.4%)		/	1(Ref)
	GT	40(48.2%)	12(70.6%)		0.176	0.45(0.14,1.43)
	GG	6(7.2%)	0(0%)		NA	NA
	GT+GG vs.TT	/	/		0.258	0.52(0.17,1.62)
	GG vs.GT+TT	/	/		NA	NA
IL10	rs1800896	N=84	N=17	0.806		
	TT	72(85.7%)	14(82.4%)		/	1(Ref)
	CT	11(13.1%)	3(17.6%)		0.648	0.72(0.17,2.96)
	CC	1(1.2%)	0(0%)		NA	NA
	CT+CC vs. TT	/	/		0.719	0.77(0.19,3.15)
	CC vs.CT+TT	/	/		NA	NA
IL1A	rs17561	N=84	N=17	0.764		
	CC	63(75.0%)	14(82.4%)		/	1(Ref)
	CA	20(23.8%)	3(17.6%)		0.733	1.27(0.32,5.02)
	AA	1(1.2%)	0(0%)		NA	NA
	CA+AA vs. CC	/	/		0.631	1.40(0.36,5.44)
	AA vs.CA+CC	/	/		NA	NA
IL1B	rs1143627	N=85	N=17	0.979		
	AA	19(22.4%)	4(23.5%)		/	1(Ref)
	AG	44(51.8%)	9(52.9%)		0.896	0.92(0.24,3.46)
	GG	22(25.9%)	4(23.5%)		1.000	1.00(0.21,4.79)
	AG+GG vs. AA	/	/		0.962	0.97(0.28,3.40)
	GG vs. AG+AA	/	/		0.890	1.09(0.32,3.75)
IL1B	rs16944	N=84	N=17	0.974		
	GG	19(22.6%)	4(23.5%)		/	1(Ref)
	GA	43(51.2%)	9(52.9%)		0.960	0.97(0.26,3.62)
	AA	22(26.2%)	4(23.5%)		0.956	1.05(0.22,5.00)
	GA+AA vs. GG	/	/		0.988	1.01(0.29,3.51)
	AA vs.GA+GG	/	/		0.873	1.11(0.32,3.80)
IL1B	rs1143634	N=84	N=17	0.610		
	GG	80(95.2%)	16(94.1%)		/	1(Ref)
	GA	4(4.8%)	1(5.9%)		0.927	0.90(0.09,8.89)
	AA	0(0%)	0(0%)		NA	NA
	GA+AA vs. GG	/	/		0.927	0.90(0.09,8.89)
	AA vs.GA+GG	/	/		NA	NA
IL1RN	rs419598	N=63	N=16	**0.007**		
	TT	59(93.7%)	11(68.8%)		/	1(Ref)
	CT	3(4.8%)	5(31.3%)		**0.013**	0.12(0.02,0.64)
	CC	1(1.6%)	0(0%)		NA	NA
	CT+CC vs. TT	/	/		**0.022**	0.16(0.03,0.77)
	CC vs.CT+TT	/	/		NA	NA
IL21R	rs2189521	N=85	N=17	0.404		
	TT	52(61.2%)	13(76.5%)		/	1(Ref)
	CT	29(34.1%)	4(23.5%)		0.288	1.95(0.57,6.66)
	CC	4(4.7%)	0(0%)		NA	NA
	CT+CC vs. TT	/	/		0.203	2.21(0.65,7.50)
	CC vs.CT+TT	/	/		NA	NA
IL4	rs2243250	N=85	N=17	0.446		
	CC	4(4.7%)	0(0%)		/	1(Ref)
	CT	29(34.1%)	8(47.1%)		NA	NA
	TT	52(61.2%)	9(52.9%)		NA	NA
	CT+TT vs. CC	/	/		NA	NA
	TT vs.CT+CC	/	/		0.530	1.40(0.49,4.05)
IL4	rs2227284	N=85	N=17	0.293		
	TT	60(70.6%)	9(52.9%)		/	1(Ref)
	GT	24(28.2%)	8(47.1%)		0.126	0.43(0.15,1.27)
	GG	1(1.2%)	0(0%)		NA	NA
	GT+GG vs.TT	/	/		0.141	0.44(0.15,1.31)
	GG vs.GT+TT	/	/		NA	NA
IL4RA	rs1801275	N=83	N=17	0.901		
	AA	63(75.9%)	13(76.5%)		/	1(Ref)
	GA	19(22.9%)	4(23.5%)		0.832	0.87(0.25,3.07)
	GG	1(1.2%)	0(0%)		NA	NA
	GA+GG vs. AA	/	/		0.885	0.91(0.26,3.20)
	GG vs. GA+AA	/	/		NA	NA
IL6	rs1800796	N=85	N=17	0.809		
	GG	17(20.0%)	4(23.5%)		/	1(Ref)
	CG	32(37.6%)	5(29.4%)		0.261	2.57(0.50,13.38)
	CC	36(42.4%)	8(47.1%)		0.894	1.10(0.29,4.19)
	CG+CC vs.GG	/	/		0.571	1.45(0.40,5.18)
	CC vs.CG+GG	/	/		0.634	0.77(0.27,2.24)
PIGR	rs291097	N=84	N=17	0.321		
	GG	78(92.9%)	17(100%)		/	1(Ref)
	GA	6(7.1%)	0(0.0%)		**NA**	NA
	AA	0(0%)	0(0.0%)		NA	NA
	GA+AA vs. GG	/	/		**NA**	NA
	AA vs.GA+GG	/	/		NA	NA
PIGR	rs291102	N=85	N=17	0.383		
	GG	69(81.2%)	15(88.2%)		/	1(Ref)
	GA	16(18.8%)	2(11.8%)		0.630	1.48(0.30,7.34)
	AA	0(0%)	0(0%)		NA	NA
	GA+AA vs. GG	/	/		0.630	1.48(0.30,7.34)
	AA vs.GA+GG	/	/		NA	NA
TNF	rs1799964	N=85	N=17	0.996		
	TT	51(60.0%)	10(58.8%)		/	1(Ref)
	CT	29(34.1%)	5(35.3%)		0.934	1.05(0.34,3.27)
	CC	5(5.9%)	1(5.9%)		0.925	0.89(0.09,9.22)
	CT+CC vs. TT	/	/		0.899	1.07(0.36,3.18)
	CC vs.CT+TT	/	/		0.931	0.91(0.10,8.49)
TNF	rs1800629	N=85	N=17	0.833		
	GG	0(0%)	0(0%)		/	1(Ref)
	GA	84(98.8%)	17(100%)		NA	NA
	AA	1(1.2%)	0(0%)		NA	NA
	GA+AA vs. GG	/	/		NA	NA
	AA vs.GA+GG	/	/		NA	NA
TNFRSF1A	rs4149570	N=82	N=17	0.513		
	CC	21(25.6%)	6(35.3%)		/	1(Ref)
	CA	36(43.9%)	8(47.1%)		0.569	1.42(0.42,4.81)
	AA	25(30.5%)	3(17.6%)		0.258	2.40(0.53,10.90)
	CA+AA vs. CC	/	/		0.360	1.70(0.55,5.27)
	AA vs.CA+CC	/	/		0.330	1.95(0.51,7.48)
TNFSF7	rs7259857	N=85	N=17	0.175		
	TT	63(74.1%)	15(88.2%)		/	1(Ref)
	CT	22(25.9%)	2(11.8%)		0.253	2.49(0.52,11.90)
	CC	0(0%)	0(0%)		NA	NA
	CT+CC vs. TT	/	/		0.253	2.49(0.52,11.90)
	CC vs.CT+TT	/	/		NA	NA
TNF	rs361525	N=85	N=17	0.267		
	GG	77(90.6%)	14(82.4%)		/	1(Ref)
	GA	8(9.4%)	3(17.6%)		0.452	0.57(0.13,2.49)
	AA	0(0%)	0(0%)		NA	NA
	GA+AA vs. GG	/	/		0.452	0.57(0.13,2.49)
	AA vs.GA+GG	/	/		NA	NA
TNF	rs1800630	N=85	N=17	0.731		
	CC	57(67.1%)	12(70.6%)		/	1(Ref)
	CA	25(29.4%)	5(29.4%)		0.829	1.14(0.36,3.64)
	AA	3(3.5%)	0(0%)		NA	NA
	CA+AA vs. CC	/	/		0.706	1.25(0.39,3.96)
	AA vs.CA+CC	/	/		0.327	2.06(0.49,8.75)
TNF	rs1799724	N=82	N=17	0.806		
	CC	62(75.6%)	13(76.5%)		/	1(Ref)
	CT	18(22.0%)	4(23.5%)		0.970	0.98(0.28,3.44)
	TT	2(2.4%)	0(0%)		NA	NA
	CT+TT vs. CC	/	/		0.872	1.11(0.32,3.86)
	TT vs.CT+CC	/	/		NA	NA
COX-2	rs5275	N=85	N=16	0.210		
	AA	61(71.8%)	8(50.0%)		/	1(Ref)
	GA	22(25.9%)	7(43.8%)		0.096	0.37(1.12,1.19)
	GG	2(2.4%)	1(6.3%)		0.135	0.13(0.01,1.88)
	GA+GG vs. AA	/	/		0.066	0.35(0.11,1.07)
	GG vs. GA+AA	/	/		0.285	0.25(0.02,3.13)
COX-2	rs20417	N=85	N=17	0.557		
	CC	76(89.4%)	14(82.4%)		/	1(Ref)
	CG	8(9.4%)	3(17.6%)		0.217	0.39(0.09,1.74)
	GG	1(1.2%)	0(0%)		NA	NA
	CG+GG vs.CC	/	/		0.269	0.43(0.10,1.91)
	GG vs.CG+CC	/	/		NA	NA
BCL2	rs2279115	N=85	N=17	0.355		
	GG	38(44.7%)	5(29.4%)		/	1(Ref)
	GT	34(40.0%)	10(58.8%)		0.149	0.41(0.12,1.38)
	TT	13(15.3%)	2(11.8%)		0.851	0.84(0.14,4.96)
	GT+TT vs.GG	/	/		0.228	0.50(0.16,1.55)
	TT vs.GT+GG	/	/		0.703	1.37(0.27,6.80)
Age≤60						
AKT1	rs1130233	N=124	N=383	**0.031**		
	CC	37(29.8%)	75(19.6%)		/	1(Ref)
	CT	57(46.0%)	181(47.3%)		0.007	0.64(0.39,1.05)
	TT	30(24.2%)	127(33.2%)		**0.014**	0.48(0.27,0.86)
	CT+TT vs. CC	/	/		**0.021**	0.57(0.36,0.92)
	TT vs.CT+CC	/	/		0.080	0.66(0.41,1.05)
AKT1	rs2494732	N=124	N=378	0.212		
	TT	11(8.9%)	27(7.1%)		/	1(Ref)
	CT	58(46.8%)	149(39.4%)		0.765	0.89(0.41,1.93)
	CC	55(44.4%)	202(53.4%)		0.191	0.59(0.27,1.30)
	CT+CC vs. TT	/	/		0.379	0.71(0.34,1.51)
	CC vs.CT+TT	/	/		0.085	0.69(0.46,1.05)
CR2	rs3813946	N=124	N=379	0.497		
	TT	92(74.2%)	299(78.9%)		/	1(Ref)
	CT	31(25.0%)	76(20.1%)		0.333	1.27(0.78,2.07)
	CC	1(0.8%)	4(1.1%)		0.749	0.70(0.08,6.40)
	CT+CC vs. TT	/	/		0.382	1.24(0.77,2.00)
	CC vs.CT+TT	/	/		0.694	0.64(0.70,5.90)
IL10	rs1800871	N=125	N=383	0.913		
	AA	53(42.4%)	159(41.5%)		/	1(Ref)
	GA	58(46.4%)	185(48.3%)		0.801	0.95(0.61,1.46)
	GG	14(11.2%)	39(10.2%)		0.996	1.00(0.50,2.00)
	GA+GG vs. AA	/	/		0.825	0.95(0.63,1.45)
	GG vs. GA+AA	/	/		0.943	1.02(0.53,1.98)
IL10	rs1800872	N=125	N=383	0.913		
	TT	53(42.4%)	159(41.5%)		/	1(Ref)
	GT	58(46.4%)	185(48.3%)		0.801	0.95(0.61,1.46)
	GG	14(11.2%)	39(10.2%)		0.996	1.00(0.50,2.00)
	GT+GG vs.TT	/	/		0.825	0.95(0.63,1.45)
	GG vs.GT+TT	/	/		0.943	1.02(0.53,1.98)
IL10	rs1800896	N=125	N=383	0.651		
	TT	102(81.6%)	308(80.4%)		/	1(Ref)
	CT	22(17.6%)	74(19.3%)		0.504	0.83(0.49,1.42)
	CC	1(0.8%)	1(0.3%)		0.382	3.62(0.20,64.97)
	CT+CC vs. TT	/	/		0.582	0.86(0.51,1.46)
	CC vs.CT+TT	/	/		0.374	0.67(0.21,64.70)
IL1A	rs17561	N=124	N=383	0.932		
	CC	103(83.1%)	313(81.7%)		/	1(Ref)
	CA	20(16.1%)	66(17.2%)		0.785	0.93(0.53,1.62)
	AA	1(0.8%)	4(1.0%)		0.679	0.63(0.07,5.79)
	CA+AA vs. CC	/	/		0.725	0.91(0.53,1.56)
	AA vs.CA+CC	/	/		0.703	0.65(0.07,5.98)
IL1B	rs1143627	N=123	N=377	0.768		
	AA	32(26.0%)	107(28.4%)		/	1(Ref)
	AG	63(51.2%)	179(47.5%)		0.550	1.16(0.71,1.91)
	GG	28(22.8%)	91(24.1%)		0.950	1.02(0.57,1.83)
	AG+GG vs. AA	/	/		0.654	1.11(0.70,1.78)
	GG vs. AG+AA	/	/		0.739	0.92(0.56,1.50)
IL1B	rs16944	N=125	N=380	0.883		
	GG	33(26.4%)	107(28.2%)		/	1(Ref)
	GA	63(50.4%)	182(47.9%)		0.678	1.11(0.68,1.82)
	AA	29(23.2%)	91(23.9%)		0.953	1.02(0.57,1.81)
	GA+AA vs. GG	/	/		0.755	1.08(0.68,1.71)
	AA vs.GA+GG	/	/		0.819	0.95(0.58,1.54)
IL1B	rs1143634	N=125	N=383	0.838		
	GG	119(95.2%)	365(95.3%)		/	1(Ref)
	GA	6(4.8%)	17(4.4%)		0.858	1.09(0.41,2.89)
	AA	0(0%)	1(0.3%)		NA	NA
	GA+AA vs. GG	/	/		0.913	1.06(0.40,2.77)
	AA vs.GA+GG	/	/		NA	NA
IL1RN	rs419598	N=80	N=377	0.919		
	TT	69(86.3%)	325(86.2%)		/	1(Ref)
	CT	10(12.5%)	49(13.0%)		0.870	1.06(0.51,2.23)
	CC	1(1.3%)	3(0.8%)		0.764	1.42(0.14,14.18)
	CT+CC vs. TT	/	/		0.815	1.09(0.53,2.22)
	CC vs.CT+TT	/	/		0.776	1.40(0.14,13.97)
IL21R	rs2189521	N=123	N=378	**0.049**		
	TT	79(64.2%)	195(51.6%)		/	1(Ref)
	CT	38(30.9%)	156(41.3%)		**0.031**	0.61(0.39,0.96)
	CC	6(4.9%)	27(7.1%)		0.208	0.55(0.22,1.39)
	CT+CC vs. TT	/	/		**0.019**	0.60(0.39,0.92)
	CC vs.CT+TT	/	/		0.381	0.66(0.26,1.67)
IL4	rs2243250	N=124	N=378	0.371		
	CC	5(4.0%)	13(3.4%)		/	1(Ref)
	CT	47(37.9%)	119(31.5%)		0.922	0.95(0.31,2.88)
	TT	72(58.1%)	246(65.1%)		0.558	0.72(0.25,2.14)
	CT+TT vs. CC	/	/		0.676	0.80(0.27,2.33)
	TT vs.CT+CC	/	/		0.189	0.75(0.49,1.15)
IL4	rs2227284	N=124	N=378	0.216		
	TT	84(67.7%)	285(75.4%)		/	1(Ref)
	GT	36(29.0%)	86(22.8%)		0.323	1.27(0.79,2.02)
	GG	4(3.2%)	7(1.9%)		0.266	2.08(0.57,7.59)
	GT+GG vs.TT	/	/		0.231	1.32(0.84,2.07)
	GG vs.GT+TT	/	/		0.310	1.94(0.54,6.99)
IL4RA	rs1801275	N=124	N=383	0.239		
	AA	89(71.8%)	259(67.6%)		/	1(Ref)
	GA	34(27.4%)	110(28.7%)		0.870	0.96(0.61,1.53)
	GG	1(0.8%)	14(3.7%)		0.165	0.23(0.03,1.82)
	GA+GG vs. AA	/	/		0.597	0.88(0.56,1.40)
	GG vs. GA+AA	/	/		0.170	0.24(0.03,1.85)
IL6	rs1800796	N=124	N=378	0.411		
	GG	9(7.3%)	43(11.4%)		/	1(Ref)
	CG	55(44.4%)	165(43.7%)		0.444	1.37(0.61,3.07)
	CC	60(48.4%)	170(45.0%)		0.281	1.54(0.70,3.38)
	CG+CC vs.GG	/	/		0.338	1.45(0.68,3.11)
	CC vs.CG+GG	/	/		0.616	1.11(0.74,1.68)
PIGR	rs291097	N=125	N=383	0.077		
	GG	110(88.0%)	355(92.7%)		/	1(Ref)
	GA	15(12.0%)	28(7.3%)		0.108	1.74(0.86,3.44)
	AA	0(0.0%)	0(0.0%)		NA	NA
	GA+AA vs. GG	/	/		0.108	1.74(0.86,3.44)
	AA vs.GA+GG	/	/		NA	NA
PIGR	rs291102	N=123	N=379	0.591		
	GG	96(78.0%)	308(81.3%)		/	1(Ref)
	GA	25(20.3%)	68(17.9%)		0.376	1.27(0.75,2.14)
	AA	2(1.6%)	3(0.8%)		NA	NA
	GA	/	/		0.284	1.32(0.79,2.21)
	AA vs.GA+GG	/	/		NA	NA
TNF	rs1799964	N=124	N=378	0.775		
	TT	73(58.9%)	236(62.4%)		/	1(Ref)
	CT	45(36.3%)	126(33.3%)		0.537	1.15(0.74,1.78)
	CC	6(4.8%)	16(4.2%)		0.666	1.25(0.46,3.38)
	CT+CC vs. TT	/	/		0.493	1.16(0.76,1.77)
	CC vs.CT+TT	/	/		0.740	1.18(0.44,3.15)
TNF	rs1800629	N=124	N=379	0.567		
	GG	0(0%)	0(0%)		/	1(Ref)
	GA	124(100%)	377(99.5%)		NA	NA
	AA	0(0%)	2(0.5%)		NA	NA
	GA+AA vs. GG	/	/		NA	NA
	AA vs.GA+GG	/	/		NA	NA
TNFRSF1A	rs4149570	N=123	N=378	0.256		
	CC	22(17.9%)	95(25.1%)		/	1(Ref)
	CA	66(53.7%)	186(49.2%)		0.204	1.43(0.82,2.50)
	AA	35(28.5%)	97(25.7%)		0.157	1.55(0.85,2.84)
	CA+AA vs. CC	/	/		0.142	1.48(0.88,2.50)
	AA vs.CA+CC	/	/		0.468	1.19(0.75,1.89)
TNFSF7	rs7259857	N=124	N=379	0.379		
	TT	103(83.1%)	307(81.0%)		/	1(Ref)
	CT	18(14.5%)	68(17.9%)		0.347	0.76(0.43,1.35)
	CC	3(2.4%)	4(1.1%)		NA	NA
	CT+CC vs. TT	/	/		0.539	0.84(0.49,1.45)
	CC vs.CT+TT	/	/		NA	NA
TNF	rs361525	N=124	N=379	0.506		
	GG	114(91.9%)	350(92.3%)		/	1(Ref)
	GA	10(8.1%)	29(7.7%)		0.957	0.98(0.46,2.10)
	AA	0(0%)	0(0%)		NA	NA
	GA+AA vs. GG	/	/		0.957	0.98(0.46,2.10)
	AA vs.GA+GG	/	/		NA	NA
TNF	rs1800630	N=122	N=378	0.978		
	CC	84(68.9%)	262(69.3%)		/	1(Ref)
	CA	34(27.9%)	105(27.8%)		0.824	1.06(0.66,1.69)
	AA	4(3.3%)	11(2.9%)		0.795	1.17(0.35,3.90)
	CA+AA vs. CC	/	/		0.797	1.06(0.68,1.66)
	AA vs.CA+CC	/	/		0.327	2.06(0.49,8.75)
TNF	rs1799724	N=123	N=381	0.915		
	CC	91(74.0%)	289(75.9%)		/	1(Ref)
	CT	30(24.4%)	86(22.6%)		0.737	1.09(0.67,1.77)
	TT	2(1.6%)	6(1.5%)		0.930	0.93(0.18,4.79)
	CT+TT vs. CC	/	/		0.764	1.08(0.67,1.73)
	CT+TT vs. CC	/	/		0.908	0.91(1.18,4.67)
COX-2	rs5275	N=124	N=380	0.418		
	AA	78(62.9%)	262(68.9%)		/	1(Ref)
	GA	43(34.7%)	108(28.4%)		0.310	1.26(0.81,1.96)
	GG	3(2.4%)	10(2.6%)		0.978	0.98(0.26,3.75)
	GA+GG vs. AA	/	/		0.343	1.23(0.80,1.90)
	GG vs. GA+AA	/	/		0.893	0.91(0.24,3.45)
COX-2	rs20417	N=123	N=376	0.826		
	CC	112(91.1%)	344(91.5%)		/	1(Ref)
	CG	11(8.9%)	31(8.2%)		0.968	0.99(0.47,2.05)
	GG	0(0.0%)	1(0.3%)		NA	NA
	CG+GG vs.CC	/	/		0.929	0.97(0.47,2.01)
	GG vs.CG+CC	/	/		NA	NA
BCL2	rs2279115	N=124	N=378	0.276		
	GG	58(46.8%)	161(42.6%)		/	1(Ref)
	GT	54(43.5%)	159(42.1%)		0.920	1.02(0.66,1.59)
	TT	12(9.7%)	58(15.3%)		0.057	0.51(0.25,1.02)
	GT+TT vs.GG	/	/		0.462	0.86(0.57,1.30)
	TT vs.GT+GG	/	/		**0.037**	0.49(0.25,0.96)
Male						
AKT1	rs1130233	N=160	N=225	**0.028**		
	CC	48(30.0%)	42(18.7%)		/	1(Ref)
	CT	71(44.4%)	109(48.4%)		0.088	0.49(0.21,1.11)
	TT	41(25.6%)	74(32.9%)		**0.014**	0.37(0.17,0.82)
	CT+TT vs. CC	/	/		**0.025**	0.43(0.21,0.90)
	TT vs.CT+CC	/	/		0.062	0.53(0.28,1.03)
AKT1	rs2494732	N=161	N=222	0.516		
	TT	13(8.1%)	13(5.9%)		/	1(Ref)
	CT	72(44.7%)	93(41.9%)		0.249	0.44(0.11,1.79)
	CC	76(47.2%)	116(52.3%)		0.143	0.37(0.10,1.40)
	CT+CC vs. TT	/	/		0.175	0.40(0.11,1.50)
	CC vs.CT+TT	/	/		0.292	0.73(0.40,1.32)
CR2	rs3813946	N=161	N=222	0.226		
	TT	115(71.4%)	175(78.8%)		/	1(Ref)
	CT	44(27.3%)	44(19.8%)		0.915	1.04(0.52,2.07)
	CC	2(1.2%)	3(1.4%)		0.152	0.21(0.02,1.78)
	CT+CC vs. TT	/	/		0.829	0.93(0.47,1.82)
	CC vs.CT+TT	/	/		0.130	0.19(0.02,1.64)
IL10	rs1800871	N=160	N=225	0.876		
	AA	68(42.5%)	92(40.9%)		/	1(Ref)
	GA	76(47.5%)	107(47.6%)		0.561	0.83(0.45,1.54)
	GG	16(10.0%)	26(11.6%)		0.481	1.49(0.49,4.48)
	GA+GG vs. AA	/	/		0.741	0.90(0.50,1.65)
	GG vs. GA+AA	/	/		0.355	1.63(0.58,4.55)
IL10	rs1800872	N=160	N=225	0.876		
	TT	68(42.5%)	92(40.9%)		/	1(Ref)
	GT	76(47.5%)	107(47.6%)		0.561	0.83(0.45,1.54)
	GG	16(10.0%)	26(11.6%)		0.481	1.49(0.49,4.48)
	GT+GG vs.TT	/	/		0.741	0.90(0.50,1.65)
	GG vs.GT+TT	/	/		0.355	1.63(0.58,4.55)
IL10	rs1800896	N=161	N=225	0.070		
	TT	134(83.2%)	175(77.8%)		/	1(Ref)
	CT	25(15.5%)	50(22.2%)		0.227	0.63(0.30,1.33)
	CC	2(1.2%)	0(0%)		NA	NA
	CT+CC vs. TT	/	/		0.305	0.68(0.32,1.42)
	CC vs.CT+TT	/	/		NA	NA
IL1A	rs17561	N=160	N=225	0.237		
	CC	130(81.3%)	179(79.6%)		/	1(Ref)
	CA	30(18.8%)	42(18.7%)		0.860	0.93(0.42,2.09)
	AA	0(0%)	4(1.8%)		NA	NA
	CA+AA vs. CC	/	/		0.692	0.85(0.39,1.88)
	AA vs.CA+CC	/	/		NA	NA
IL1B	rs1143627	N=160	N=220	0.281		
	AA	35(21.9%)	63(28.6%)		/	1(Ref)
	AG	86(53.8%)	103(46.8%)		0.280	1.47(0.73,2.95)
	GG	39(24.4%)	54(24.5%)		0.807	1.12(0.46,2.75)
	AG+GG vs. AA	/	/		0.360	1.36(0.70,2.65)
	GG vs. AG+AA	/	/		0.789	0.91(0.44,1.86)
IL1B	rs16944	N=161	N=223	0.475		
	GG	37(23.0%)	63(28.3%)		/	1(Ref)
	GA	84(52.2%)	105(47.1%)		0.345	1.40(0.70,2.79)
	AA	40(24.8%)	55(24.7%)		0.724	1.17(0.48,2.86)
	GA+AA vs. GG	/	/		0.395	1.33(0.69,2.57)
	AA vs.GA+GG	/	/		0.933	0.97(0.48,1.97)
IL1B	rs1143634	N=161	N=225	0.388		
	GG	155(96.3%)	214(95.1%)		/	1(Ref)
	GA	6(3.7%)	11(4.9%)		0.979	0.98(0.23,4.11)
	AA	0(0.0%)	0(0.0%)		NA	NA
	GA+AA vs. GG	/	/		0.979	0.98(0.23,4.11)
	AA vs.GA+GG	/	/		NA	NA
IL1RN	rs419598	N=109	N=220	0.972		
	TT	98(89.9%)	196(89.1%)		/	1(Ref)
	CT	10(9.2%)	22(10.0%)		0.878	0.92(0.29,2.87)
	CC	1(0.9%)	2(0.9%)		0.638	1.88(0.14,26.09)
	CT+CC vs. TT	/	/		0.994	1.00(0.34,2.95)
	CC vs.CT+TT	/	/		0.659	1.80(0.13,24.45)
IL21R	rs2189521	N=160	N=222	0.364		
	TT	98(61.3%)	123(55.4%)		/	1(Ref)
	CT	55(34.4%)	83(37.4%)		0.631	1.17(0.62,2.21)
	CC	7(4.4%)	16(7.2%)		0.703	0.78(0.22,2.79)
	CT+CC vs. TT	/	/		0.748	1.11(0.60,2.03)
	CC vs.CT+TT	/	/		0.636	0.74(0.21,2.60)
IL4	rs2243250	N=161	N=222	0.736		
	CC	6(3.7%)	7(3.2%)		/	1(Ref)
	CT	59(36.6%)	74(33.3%)		0.855	0.86(0.17,4.35)
	TT	96(59.6%)	141(63.5%)		0.740	0.72(0.10,5.13)
	CT+TT vs. CC	/	/		0.770	0.77(0.13,4.42)
	TT vs.CT+CC	/	/		0.550	0.83(0.45,1.53)
IL4	rs2227284	N=161	N=222	0.715		
	TT	110(68.3%)	154(69.4%)		/	1(Ref)
	GT	47(29.2%)	65(29.3%)		0.988	1.00(0.52,1.91)
	GG	4(2.5%)	3(1.4%)		0.372	3.91(0.20,78.06)
	GT+GG vs.TT	/	/		0.872	1.05(0.55,2.01)
	GG vs.GT+TT	/	/		0.366	3.36(0.24,46.79)
IL4RA	rs1801275	N=159	N=225	0.609		
	AA	114(71.7%)	162(72.0%)		/	1(Ref)
	GA	43(27.0%)	57(25.3%)		0.745	1.17(1.13,1.20)
	GG	2(1.3%)	6(2.7%)		0.638	0.59(0.06,5.44)
	GA+GG vs. AA	/	/		0.831	1.07(0.56,2.07)
	GG vs. GA+AA	/	/		0.605	0.54(0.05,5.50)
IL6	rs1800796	N=161	N=222	0.566		
	GG	21(13.0%)	22(9.9%)		/	1(Ref)
	CG	68(42.2%)	92(41.4%)		0.665	1.26(0.44,3.63)
	CC	72(44.7%)	108(48.6%)		0.856	1.10(0.39,3.08)
	CG+CC vs.GG	/	/		0.740	1.18(0.44,3.20)
	CC vs.CG+GG	/	/		0.804	0.93(0.51,1.68)
PIGR	rs291097	N=161	N=225	0.331		
	GG	146(90.7%)	208(92.4%)		/	1(Ref)
	GA	15(9.3%)	17(7.6%)		0.245	1.89(0.65,5.49)
	AA	0(0.0%)	0(0.0%)		NA	NA
	GA+AA vs. GG	/	/		0.245	1.89(0.65,5.49)
	AA vs.GA+GG	/	/		NA	NA
PIGR	rs291102	N=160	N=222	0.613		
	GG	127(79.4%)	185(83.3%)		/	1(Ref)
	GA	32(20.0%)	36(16.2%)		0.304	1.51(0.69,3.31)
	AA	1(0.6%)	1(0.5%)		0.889	1.33(0.02,74.43)
	GA+AA vs. GG	/	/		0.301	1.51(0.69,3.27)
	AA vs.GA+GG	/	/		0.920	1.22(0.02,62.17)
TNF	rs1799964	N=161	N=221	0.904		
	TT	101(62.7%)	135(61.1%)		/	1(Ref)
	CT	52(32.3%)	76(34.4%)		0.693	1.14(0.61,2.13)
	CC	8(5.0%)	10(4.5%)		0.728	1.38(0.22,8.52)
	CT+CC vs. TT	/	/		0.646	1.16(0.63,2.13)
	CC vs.CT+TT	/	/		0.758	1.31(0.23,7.37)
TNF	rs1800629	N=161	N=221	0.413		
	GG	101(62.7%)	135(61.1%)		/	1(Ref)
	GA	0(0%)	0(0%)		NA	NA
	AA	60(37.3%)	86(38.9%)		NA	NA
	GA+AA vs. GG	/	/		NA	NA
	AA vs.GA+GG	/	/		0.541	0.38(0.02,8.39)
TNFRSF1A	rs4149570	N=158	N=225	0.422		
	CC	31(19.6%)	57(25.3%)		/	1(Ref)
	CA	84(53.2%)	110(48.9%)		0.065	2.14(0.96,4.81)
	AA	43(27.2%)	58(25.8%)		0.300	1.62(0.65,4.01)
	CA+AA vs. CC	/	/		0.090	1.92(0.90,4.10)
	AA vs.CA+CC	/	/		0.817	0.92(0.47,1.82)
TNFSF7	rs7259857	N=161	N=222	0.832		
	TT	126(78.3%)	179(80.6%)		/	1(Ref)
	CT	33(20.5%)	41(18.5%)		0.905	1.05(0.48,2.31)
	CC	2(1.2%)	2(0.9%)		0.283	3.86(0.33,45.27)
	CT+CC vs. TT	/	/		0.695	1.16(0.55,2.48)
	CC vs.CT+TT	/	/		0.276	3.95(0.33,46.85)
TNF	rs361525	N=161	N=222	0.406		
	GG	146(90.7%)	204(91.9%)		/	1(Ref)
	GA	15(9.3%)	18(8.1%)		0.053	3.05(0.99,9.42)
	AA	0(0%)	0(0%)		NA	NA
	GA+AA vs. GG	/	/		0.053	3.05(0.98,9.42)
	AA vs.GA+GG	/	/		NA	NA
TNF	rs1800630	N=159	N=222	0.708		
	CC	115(72.3%)	153(68.9%)		/	1(Ref)
	CA	40(25.2%)	61(27.5%)		0.462	0.78(0.40,1.52)
	AA	4(2.5%)	8(3.6%)		0.478	0.46(0.05,3.95)
	CA+AA vs. CC	/	/		0.392	0.75(0.39,1.45)
	AA vs.CA+CC	/	/		0.520	0.50(0.06,4.18)
TNF	rs1799724	N=160	N=223	0.997		
	CC	120(75.0%)	167(74.9%)		/	1(Ref)
	CT	37(23.1%)	52(23.3%)		0.777	1.11(0.55,2.23)
	TT	3(1.9%)	4(1.8%)		0.703	1.97(0.06,63.57)
	CT+TT vs. CC	/	/		0.733	1.13(0.56,2.26)
	TT vs.CT+CC	/	/		0.714	1.90(0.06,58.99)
COX-2	rs5275	N=161	N=222	0.965		
	AA	105(65.2%)	144(64.9%)		/	1(Ref)
	GA	51(31.7%)	72(32.4%)		0.902	1.04(0.54,1.99)
	GG	5(3.1%)	6(2.7%)		0.800	1.37(0.12,15.44)
	GA+GG vs. AA	/	/		0.867	1.06(0.56,1.99)
	GG vs. GA+AA	/	/		0.808	1.32(0.14,12.27)
COX-2	rs20417	N=160	N=219	0.503		
	CC	142(88.8%)	196(89.5%)		/	1(Ref)
	CG	17(10.6%)	23(10.5%)		0.607	0.77(0.28,2.12)
	GG	1(0.6%)	0(0%)		NA	NA
	CG+GG vs.CC	/	/		0.618	0.77(0.28,2.13)
	GG vs.CG+CC	/	/		NA	NA
BCL2	rs2279115	N=161	N=222	**0.036**		
	GG	75(46.6%)	93(41.9%)		/	1(Ref)
	GT	68(42.2%)	82(36.9%)		0.824	0.93(0.49,1.75)
	TT	18(11.2%)	47(21.2%)		**0.044**	0.37(0.14,0.97)
	GT+TT vs.GG	/	/		0.349	0.75(0.41,1.37)
	TT vs.GT+GG	/	/		**0.044**	0.41(0.17,0.98)
Female						
AKT1	rs1130233	N=48	N=175	0.299		
	CC	10(20.8%)	35(20.0%)		/	1(Ref)
	CT	27(56.3%)	80(45.7%)		0.914	1.05(0.42,2.66)
	TT	11(22.9%)	60(34.3%)		0.457	0.66(0.22,2.00)
	CT+TT vs. CC	/	/		0.792	0.89(0.36,2.17)
	TT vs.CT+CC	/	/		0.288	0.64(0.28,1.46)
AKT1	rs2494732	N=48	N=173	0.118		
	TT	5(10.4%)	14(8.1%)		/	1(Ref)
	CT	25(52.1%)	65(37.6%)		0.716	0.79(0.22,2.81)
	CC	18(37.5%)	94(54.3%)		0.183	0.40(0.10,1.54)
	CT+CC vs. TT	/	/		0.370	0.57(0.16,1.96)
	CC vs.CT+TT	/	/		0.098	0.54(0.26,1.12)
CR2	rs3813946	N=48	N=174	0.848		
	TT	39(81.3%)	138(79.3%)		/	1(Ref)
	CT	9(18.8%)	35(20.1%)		0.560	0.75(0.29,1.95)
	CC	0(0%)	1(0.6%)		NA	NA
	CT+CC vs. TT	/	/		0.504	0.73(0.28,1.86)
	CC vs.CT+TT	/	/		NA	NA
IL10	rs1800871	N=48	N=175	0.790		
	AA	22(45.8%)	72(41.1%)		/	1(Ref)
	GA	22(45.8%)	90(51.4%)		0.472	0.76(0.36,1.61)
	GG	4(8.3%)	13(7.4%)		0.963	1.03(0.27,4.00)
	GA+GG vs. AA	/	/		0.525	0.79(0.38,1.64)
	GG vs. GA+AA	/	/		0.786	1.19(0.33,4.28)
IL10	rs1800872	N=48	N=175	0.790		
	TT	22(45.8%)	72(41.1%)		/	1(Ref)
	GT	22(45.8%)	90(51.4%)		0.472	0.76(0.36,1.61)
	GG	4(8.3%)	13(7.4%)		0.963	1.03(0.27,4.00)
	GT+GG vs.TT	/	/		0.525	0.79(0.38,1.64)
	GG vs.GT+TT	/	/		0.783	1.19(0.33,4.28)
IL10	rs1800896	N=48	N=175	0.855		
	TT	40(83.3%)	147(84.0%)		/	1(Ref)
	CT	8(16.7%)	27(15.4%)		0.583	1.31(0.50,3.47)
	CC	0(0%)	1(0.6%)		NA	NA
	CT+CC vs. TT	/	/		0.668	1.24(0.47,3.24)
	CC vs.CT+TT	/	/		NA	NA
IL1A	rs17561	N=48	N=175	**0.015**		
	CC	36(75.0%)	148(84.6%)		/	1(Ref)
	CA	10(20.8%)	27(15.4%)		0.440	1.44(0.57,3.61)
	AA	2(4.2%)	0(0%)		NA	NA
	CA+AA vs. CC	/	/		0.264	1.66(0.68,4.02)
	AA vs.CA+CC	/	/		NA	NA
IL1B	rs1143627	N=48	N=174	0.725		
	AA	16(33.3%)	48(27.6%)		/	1(Ref)
	AG	21(43.8%)	85(48.9%)		0.126	0.52(0.22,1.20)
	GG	11(22.9%)	41(23.6%)		0.112	0.44(0.16,1.21)
	AG+GG vs. AA	/	/		0.060	0.46(0.20,1.03)
	GG vs. AG+AA	/	/		0.322	0.64(0.27,1.54)
IL1B	rs16944	N=48	N=174	0.870		
	GG	15(31.3%)	48(27.6%)		/	1(Ref)
	GA	22(45.8%)	86(49.4%)		0.157	0.54(0.23,1.27)
	AA	11(22.9%)	40(23.0%)		0.148	0.47(0.17,1.31)
	GA+AA vs. GG	/	/		0.079	0.48(0.21,1.09)
	AA vs.GA+GG	/	/		0.359	0.66(0.28,1.59)
IL1B	rs1143634	N=48	N=175	0.414		
	GG	44(91.7%)	167(95.4%)		/	1(Ref)
	GA	4(8.3%)	7(4.0%)		0.545	1.61(0.34,7.62)
	AA	0(0.0%)	1(0.6%)		NA	NA
	GA+AA vs. GG	/	/		0.563	1.57(0.34,7.32)
	AA vs.GA+GG	/	/		NA	NA
IL1RN	rs419598	N=34	N=173	0.183		
	TT	30(88.2%)	140(80.9%)		/	1(Ref)
	CT	3(8.8%)	32(18.5%)		0.060	0.25(0.06,1.06)
	CC	1(2.9%)	1(0.6%)		0.787	1.56(0.06,39.87)
	CT+CC vs. TT	/	/		0.083	0.32(0.09,1.16)
	CC vs.CT+TT	/	/		0.663	2.01(0.09,46.98)
IL21R	rs2189521	N=48	N=173	**0.044**		
	TT	33(68.8%)	85(49.1%)		/	1(Ref)
	CT	12(25.0%)	77(44.5%)		**0.022**	0.39(0.17,0.87)
	CC	3(6.3%)	11(6.4%)		0.760	0.79(0.17,3.59)
	CT+CC vs. TT	/	/		**0.030**	0.43(0.20,0.92)
	CC vs.CT+TT	/	/		0.860	1.15(0.24,5.56)
IL4	rs2243250	N=48	N=173	0.517		
	CC	3(6.3%)	6(3.5%)		/	1(Ref)
	CT	17(35.4%)	53(30.6%)		0.609	0.63(0.11,3.73)
	TT	28(58.3%)	114(65.9%)		0.360	0.43(0.07,2.65)
	CT+TT vs. CC	/	/		0.432	0.50(0.09,2.85)
	TT vs.CT+CC	/	/		0.281	0.66(0.32,1.40)
IL4	rs2227284	N=48	N=173	0.272		
	TT	34(70.8%)	140(80.9%)		/	1(Ref)
	GT	13(27.1%)	29(16.8%)		0.113	2.03(0.85,4.85)
	GG	1(2.1%)	4(2.3%)		0.697	1.69(0.12,23.95)
	GT+GG vs.TT	/	/		0.111	2.00(0.85,4.67)
	GG vs.GT+TT	/	/		0.798	1.40(0.11,18.01)
IL4RA	rs1801275	N=48	N=175	0.066		
	AA	38(79.2%)	110(62.9%)		/	1(Ref)
	GA	10(20.8%)	57(32.6%)		0.450	0.72(0.31,1.68)
	GG	0(0%)	8(4.6%)		NA	NA
	GA+GG vs. AA	/	/		0.265	0.62(0.27,1.43)
	GG vs. GA+AA	/	/		NA	NA
IL6	rs1800796	N=48	N=173	0.469		
	GG	5(10.4%)	25(14.5%)		/	1(Ref)
	CG	19(39.6%)	78(45.1%)		0.996	1.00(0.29,3.40)
	CC	24(50.0%)	70(40.5%)		0.330	1.85(0.54,6.37)
	CG+CC vs.GG	/	/		0.613	1.35(0.42,4.33)
	CC vs.CG+GG	/	/		0.102	1.86(0.89,3.90)
PIGR	rs291097	N=48	N=175	0.131		
	GG	42(87.5%)	164(93.7%)		/	1(Ref)
	GA	6(12.5%)	11(6.3%)		**0.042**	3.43(1.05,11.23)
	AA	0(0.0%)	0(0.0%)		NA	NA
	GA+AA vs.GG	/	/		**0.042**	3.43(1.05,11.23)
	AA vs.GA+GG	/	/		NA	NA
PIGR	rs291102	N=48	N=174	0.880		
	GG	38(79.2%)	138(79.3%)		/	1(Ref)
	GA	9(18.8%)	34(19.5%)		0.123	2.15(0.81,5.67)
	AA	1(2.1%)	2(1.1%)		0.253	5.18(0.31,87.06)
	GA+AA vs. GG	/	/		0.094	2.22(0.87,5.63)
	AA vs.GA+GG	/	/		0.302	4.27(0.27,67.58)
TNF	rs1799964	N=48	N=174	0.137		
	TT	23(47.9%)	111(63.8%)		/	1(Ref)
	CT	22(45.8%)	56(32.2%)		0.261	1.55(0.72,3.33)
	CC	3(6.3%)	7(4.0%)		0.215	3.00(0.53,17.02)
	CT+CC vs. TT	/	/		0.175	1.66(0.80,3.45)
	CC vs.CT+TT	/	/		0.296	2.43(0.46,12.89)
TNF	rs1800629	N=48	N=174	**0.035**		
	GG	23(47.9%)	111(63.8%)		/	1(Ref)
	GA	0(0%)	0(0%)		NA	NA
	AA	25(52.1%)	63(36.2%)		NA	NA
	GA+AA vs. GG	/	/		NA	NA
	AA vs.GA+GG	/	/		NA	NA
TNFRSF1A	rs4149570	N=47	N=170	0.253		
	CC	12(25.5%)	44(25.9%)		/	1(Ref)
	CA	18(38.3%)	84(49.4%)		0.344	0.63(0.24,1.63)
	AA	17(36.2%)	42(24.7%)		0.556	1.32(0.53,3.31)
	CA+AA vs. CC	/	/		0.728	0.86(0.37,2.01)
	AA vs.CA+CC	/	/		0.163	1.74(0.80,3.80)
TNFSF7	rs7259857	N=48	N=174	0.840		
	TT	40(83.3%)	143(82.2%)		/	1(Ref)
	CT	7(14.6%)	29(16.7%)		0.832	0.89(0.31,2.54)
	CC	1(2.1%)	2(1.1%)		0.578	2.07(0.16,27.08)
	CT+CC vs. TT	/	/		0.969	0.98(0.36,2.63)
	CC vs.CT+TT	/	/		0.578	2.06(0.16,26.36)
TNF	rs361525	N=48	N=174	0.478		
	GG	45(93.8%)	160(92.0%)		/	1(Ref)
	GA	3(6.3%)	4(8.0%)		0.227	0.41(0.10,1.74)
	AA	0(0%)	0(0%)		NA	NA
	GA+AA vs. GG	/	/		0.227	0.41(0.10,1.74)
	AA vs.GA+GG	/	/		NA	NA
TNF	rs1800630	N=48	N=173	0.056		
	CC	26(54.2%)	121(69.9%)		/	1(Ref)
	CA	19(39.6%)	49(28.3%)		0.141	1.81(0.82,3.97)
	AA	3(6.3%)	3(1.7%)		**0.036**	9.42(1.16,76.25)
	CA+AA vs. CC	/	/		0.059	2.075(0.97,4.40)
	AA vs.CA+CC	/	/		0.056	6.71(0.95,47.39)
TNF	rs1799724	N=45	N=175	0.781		
	CC	33(73.3%)	135(77.1%)		/	1(Ref)
	CT	11(24.4%)	38(21.7%)		0.872	0.93(0.39,2.25)
	TT	1(2.2%)	2(1.1%)		0.524	2.59(0.14,48.43)
	CT+TT vs. CC	/	/		0.971	0.98(0.42,2.33)
	TT vs.CT+CC	/	/		0.513	2.75(0.13,57.01)
COX-2	rs5275	N=48	N=174	0.431		
	AA	34(70.8%)	126(72.4%)		/	1(Ref)
	GA	14(29.2%)	43(24.7%)		0.643	1.22(0.54,2.72)
	GG	0(0%)	5(2.9%)		NA	NA
	GA+GG vs. AA	/	/		0.745	1.14(0.51,2.53)
	GG vs. GA+AA	/	/		NA	NA
COX-2	rs20417	N=48	N=174	0.739		
	CC	46(95.8%)	162(93.1%)		/	1(Ref)
	CG	2(4.2%)	11(6.3%)		0.912	1.10(0.20,6.24)
	GG	0(0.0%)	1(0.6%)		NA	NA
	CG+GG vs.CC	/	/		0.932	1.08(0.19,6.05)
	GG vs.CG+CC	/	/		NA	NA
BCL2	rs2279115	N=48	N=173	0.263		
	GG	21(43.8%)	73(42.2%)		/	1(Ref)
	GT	20(41.7%)	87(50.3%)		0.899	1.05(0.49,2.25)
	TT	7(14.6%)	13(7.5%)		0.354	1.89(0.49,7.26)
	GT+TT vs.GG	/	/		0.616	1.21(0.58,2.52)
	TT vs.GT+GG	/	/		0.314	1.83(0.56,5.98)

In the group older than 60 years, the IL1RN rs419598 TT genotype was the most in the case group and the control group (P=0.007). In the subgroup younger than 60 years old, AKT1 rs1130233 CT genotype and IL21R rs2189521 TT wild-type was the most in the case group and the control group (P=0.031, P=0.049). Among men, AKT1 rs1130233 CT heterozygosity (P=0.028) and BCL2 rs2279115 GG genotype were the most (P=0.036) in the case group and the control group. Among women, IL1A rs17561 CC genotype and IL-21R rs2189521 TT genotype were the most among the case group and the control group (P=0.015, P=0.044). However, normal people with TNF rs1800629 GG genotype was the most in control group, and AA gene was the most in cases (P=0.044).In the same time, in stratified analyses, we found that the IL-1RN rs419598 TT genotype and dominance model (CT+TT vs. CC) conferred a 0.12-fold and 0.16-fold reduction in HNSCC progression, respectively, in individuals older than age 60(P=0.013, P=0.022). However, in those age 60 or younger, the AKT1 rs1130233 TT genotype and dominance model (CT+TT vs. CC) (P=0.014, P=0.021) , IL-21R rs2189521 CT genotype and dominance model (CT+ CC vs. TT) (P=0.031, P=0.019), and BCL2 rs2279115 recessive model (TT vs. GT+GG) (P=0.037) conferred a 0.48-fold, 0.57-fold, 0.61-fold, 0.60-fold, and 0.49-fold reduction in HNSCC progression, respectively. In addition, in men, the AKT1 rs1130233 TT genotype and dominance model (CT+TT vs. CC) (P=0.014, P=0.025)and the BCL2 rs2279115 TT genotype and recessive model (TT vs. GT+GG) (P=0.044, P=0.044)conferred a 0.37-fold, 0.43-fold, 0.37-fold, and 0.41-fold reduction in HNSCC progression, respectively. In women, the IL-21R rs2189521 CT genotype and dominance model (CT+TT vs. TT) conferred a 0.39-fold and 0.43-fold reduction in HNSCC progression(P=0.022, P=0.030), respectively. However, the PIGR rs291097 GA genotype and dominance model (GA+AA vs. GG) (P=0.042, P=0.042) and the TNF rs1800630 AA genotype (P=0.036) conferred a 3.43-fold, 3.43-fold, and 9.42-fold increase in HNSCC progression, respectively.

### Association of 28 Inflammation-Associated Gene SNPs With Radiotherapy Sensitivity of HNSCC Patients

We further analyzed the correlation between 28 SNPs and radiotherapy sensitivity of HNSCC individuals. We found that, compared with those with other genotypes, HNSCC patients carrying the IL-4RA rs1801275 AA wild-type genotype (40.9%) were more sensitive to radiotherapy (**Table 4**). There were no significant differences observed in the correlation analysis between the other 27 SNPs and radiotherapy sensitivity in HNSCC patients.

**Table 4:** Association of 28 inflammation-associated gene SNPs with radiotherapy sensitivity of HNSCC patients.

Genetype		Non-sensitivity	Sensitivity	P value
AKT1	rs1130233	N=17	N=28	0.363
	CC	7(15.6%)	7(15.6%)	
	CT	5(11.1%)	14(31.1%)	
	TT	5(11.1%)	7(15.6%)	
AKT1	rs2494732	N=17	N=28	0.560
	TT	2(4.4%)	16(35.6%)	
	CT	8(17.8%)	9(20.0%)	
	CC	7(15.6%)	3(6.7%)	
CR2	rs3813946	N=17	N=28	0.645
	TT	13(28.9%)	23(51.1%)	
	CT	4(8.9%)	5(11.1%)	
	CC	0(0%)	0(0%)	
IL10	rs1800871	N=16	N=28	0.809
	AA	9(20.5%)	14(31.8%)	
	GA	6(13.6%)	13(29.5%)	
	GG	1(2.3%)	1(2.3%)	
IL10	rs1800872	N=16	N=28	0.809
	TT	9(20.5%)	14(31.8%)	
	GT	6(13.6%)	13(29.5%)	
	GG	1(2.3%)	1(2.3%)	
IL10	rs1800896	N=17	N=28	0.814
	TT	15(33.3%)	24(53.3%)	
	CT	1(2.2%)	3(6.7%)	
	CC	1(2.2%)	1(2.2%)	
IL1A	rs17561	N=17	N=28	0.342
	CC	11(24.4%)	21(46.7%)	
	CA	6(13.3%)	7(15.6%)	
	AA	0(0%)	0(0%)	
IL1B	rs1143627	N=17	N=28	0.115
	AA	1(2.2%)	9(20.0%)	
	AG	11(24.4%)	14(31.1%)	
	GG	5(11.1%)	5(11.1%)	
IL1B	rs16944	N=17	N=28	0.274
	GG	2(4.4%)	9(20.0%)	
	GA	10(22.2%)	14(31.1%)	
	AA	5(11.1%)	5(11.1%)	
IL1B	rs1143634	N=17	N=28	0.316
	GG	15(33.3%)	27(60.0%)	
	GA	2(4.4%)	1(2.2%)	
	AA	0(0%)	0(0%)	
IL1RN	rs419598	N=14	N=24	0.731
	TT	13(34.2%)	21(55.3%)	
	CT	1(2.6%)	2(5.3%)	
	CC	0(0%)	1(2.6%)	
IL21R	rs2189521	N=17	N=28	0.505
	TT	11(24.4%)	18(40.0%)	
	CT	6(13.3%)	8(17.8%)	
	CC	0(0%)	2(4.4%)	
IL4	rs2243250	N=17	N=28	0.108
	CC	2(4.4%)	1(2.2%)	
	CT	10(22.2%)	10(22.2%)	
	TT	5(11.1%)	17(37.8%)	
IL4	rs2227284	N=17	N=28	0.057
	TT	6(13.3%)	20(44.4%)	
	GT	10(22.2%)	7(15.6%)	
	GG	1(2.2%)	1(2.2%)	
IL4RA	rs1801275	N=16	N=28	**0.030**
	AA	15(34.1%)	18(40.9%)	
	GA	1(2.3%)	10(22.7%)	
	GG	0(0%)	0(0%)	
IL6	rs1800796	N=17	N=28	0.814
	GG	2(4.4%)	5(11.1%)	
	CG	7(15.6%)	12(26.7%)	
	CC	8(17.8%)	11(24.4%)	
PIGR	rs291097	N=17	N=28	0.462
	GG	13(28.9%)	23(51.1%)	
	GA	4(8.9%)	5(11.1%)	
	AA	0(0%)	0(0%)	
PIGR	rs291102	N=17	N=28	0.605
	GG	12(26.7%)	20(44.4%)	
	GA	5(11.1%)	8(17.8%)	
	AA	0(0%)	0(0%)	
TNF	rs1799964	N=17	N=28	0.571
	TT	7(15.6%)	16(35.6%)	
	CT	8(17.8%)	10(22.2%)	
	CC	2(4.4%)	2(4.4%)	
TNF	rs1800629	N=17	N=28	NA
	GG	0(0%)	0(0%)	
	GA	17(37.8%)	28(62.2%)	
	AA	0(0%)	0(0%)	
TNFRSF1A	rs4149570	N=16	N=27	0.347
	CC	2(4.7%)	8(18.6%)	
	CA	8(18.6%)	13(30.2%)	
	AA	6(14.0%)	6(14.0%)	
TNFSF7	rs7259857	N=17	N=28	0.462
	TT	15(33.3%)	23(51.1%)	
	CT	2(4.4%)	5(11.1%)	
	CC	0(0%)	0(0%)	
TNF	rs361525	N=17	N=28	0.407
	GG	14(31.1%)	25(55.6%)	
	GA	3(6.7%)	3(6.7%)	
	AA	0(0%)	0(0%)	
TNF	rs1800630	N=17	N=28	0.761
	CC	10(22.2%)	19(42.2%)	
	CA	6(13.3%)	7(15.6%)	
	AA	1(2.2%)	2(4.4%)	
TNF	rs1799724	N=17	N=28	0.498
	CC	15(33.3%)	21(46.7%)	
	CT	1(2.2%)	5(11.1%)	
	TT	1(2.2%)	2(4.4%)	
COX-2	rs5275	N=17	N=28	0.496
	AA	13(28.9%)	20(44.4%)	
	GA	4(8.9%)	8(17.8%)	
	GG	0(0%)	0(0%)	
COX-2	rs20417	N=17	N=28	0.378
	CC	16(35.6%)	28(62.2%)	
	CG	1(2.2%)	0(0%)	
	GG	0(0%)	0(0%)	
BCL2	rs2279115	N=17	N=28	0.333
	GG	8(17.8%)	19(42.2%)	
	GT	7(15.6%)	6(13.3%)	
	TT	2(4.4%)	37(6.7%)	

Compared with those with other genotypes, HNSCC patients carrying the IL-4RA rs1801275 AA wild-type genotype (40.9%) were more sensitive to radiotherapy (P=0.030).

### Association of Clinicopathological Parameters With Radiotherapy Sensitivity of HNSCC Patients

We further analyzed the potential correlations between clinicopathological parameters and radiotherapy sensitivity of HNSCC patients. We found that age ≤ 60 years, non-smoker status, and normal levels of SCC were associated with increased radiotherapy sensitivity of HNSCC patients (*P*=0.033; *P*=0.033; *P*=0.030, respectively) (**Table 5**). There were no significant differences observed in the correlation analysis between other clinicopathological parameters and radiotherapy sensitivity in HNSCC patients.

**Table 5:** Association of clinicopathological parameters with radiotherapy sensitivity of HNSCC patients.

Characteristics		Non-sensitivity	Sensitivity	P value
Age				**0.033**
	Age≤60	6	19	
	Age>60	11	9	
Gender				0.277
	Female	4	11	
	Male	13	17	
T stage				0.440
	1-2	8	12	
	3-4	6	15	
N stage				0.646
	Negative	1	1	
	Positive	14	27	
M stage				0.265
	Negative	15	25	
	Positive	0	3	
Clinical stage				0.552
	I–II	2	2	
	III–IV	14	26	
Smoking				**0.033**
	No	6	19	
	Yes	11	9	
Drinking				0.384
	No	10	20	
	Yes	7	8	
Family history of cancer				0.869
	No	13	22	
	Yes	4	6	
SCC				**0.030**
	Normal	9	17	
	Increased	5	1	
CEA				0.474
	Normal	8	10	
	Increased	1	0	
CYFRA				0.197
	Normal	1	3	
	Increased	4	2	
EBV				0.800
	Negative	3	11	
	Positive	0	1	
Blood type				0.900
	A	3	6	
	B	3	3	
	AB	1	1	
	O	2	2	

We found that age ≤ 60 years, non-smoker status, and normal levels of SCC were associated with increased radiotherapy sensitivity of HNSCC patients (P=0.033; P=0.033; P=0.030, respectively).

### Association of 28 Inflammation-Associated Gene SNPs With Clinicopathological Parameters of HNSCC Patients

Among the SNPs related to the risk of HNSCC, the heterozygous and dominant model of AKT1 rs1130233 were significantly related to lymph node metastasis and non-distant metastasis. The recessive model of AKT1 rs2494732 was significantly related to male sex, stage III-IV disease, and normal carcinoembryonic antigen (CEA) levels. The IL-1RN rs419598 wild-type genotype was significantly related to stage III-IV disease, the PIGR rs291102 wild-type genotype was significantly related to normal levels of cytokeratin fragment 19 (CYFRA), and the BCL2 rs2279115 wild-type genotype was significantly related to lymph node metastasis. In addition, we found that the IL-1B rs1143627 recessive model was significantly related to normal levels of SCC, the IL-4 rs2243250 mutant, dominant model, and recessive model were significantly related to lymph node metastasis, and the IL-4 rs2227284 dominant model was significantly related to lymph node metastasis. Furthermore, the IL-6 rs1800796 heterozygous genotype and the absence of distant metastases were significantly related, whereas the mutant and recessive model were significantly related to lymph node metastasis. The IL-6 rs1800796 mutant were related to no family history of cancer and the recessive model were significantly related to stage III-IV disease. The TNFRSF1A rs414570 dominant model and recessive model were significantly related to the absence of distant metastases. The TNF rs361525 wild-type genotype was significantly related to stage III-IV disease and the COX-2 rs20417 wild-type genotype was significantly related to lymph node metastasis. The other SNPs showed no significant correlations with clinicopathological parameters. The results of association of significant inflammation-associated gene SNPs with clinicopathological parameters of HNSCC patients are shown in **Table 6**, and all results are shown in **Supplementary Table 2**.

**Table 6:** Association of significant inflammation-associated gene SNPs with clinicopathological parameters of HNSCC patients.

Characteristics		AKT1 rs1130233							AKT1 rs2494732							PIGR rs291097							PIGR rs291102						
		Wild	Heterozygous	P value	Mutation	P value	P_dominance_	P_recessive_	Wild	Heterozygous	P value	Mutation	P value	P_dominance_	P_recessive_	Wild	Heterozygous	P value	Mutation	P value	P_dominance_	P_recessive_	Wild	Heterozygous	P value	Mutation	P value	P_dominance_	P_recessive_
Age				0.675		0.661	0.634	0.806			0.649		0.862	0.740	0.724			0.117		NA	0.117	NA			0.752		0.238	0.604	0.242
	Age≤60	40	70		34				13	69		62				125	20		0				111	30		2			
	Age>60	24	48		24				40	43		44				89	7		0				78	19		0			
Gender				0.111		0.852	0.273	0.195			0.613		0.093	0.272	**0.041**			0.584		NA	0.584	NA			0.896		0.428	0.989	0.123
	Female	13	37		11				8	33		20				53	8		0				48	12		1			
	Male	51	81		47				15	79		86				161	19		0				141	37		1			
T stage				0.410		0.601	0.706	0.261			0.672		0.476	0.556	0.518			0.993		NA	0.993	NA			0.706		0.282	0.820	0.274
	1-2	28	53		20				11	49		42				89	13		0				77	24		0			
	3-4	26	37		23				7	39		39				75	11		0				66	18		1			
N stage				**0.034**		0.327	0.055	0.821			0.393		0.902	0.589	0.292			0.478		NA	0.478	NA			0.973		0.498	0.956	0.497
	Negative	12	35		13				5	34		23				55	6		0				47	14		0			
	Positive	45	58		31				14	58		60				116	18		0				102	30		1			
M stage				**0.046**		0.104	0.051	0.737			0.333		0.145	0.197	0.171			0.342		NA	0.342	NA			0.671		0.784	0.700	0.777
	Negative	57	88		43				19	91		79				167	22		0				146	41		1			
	Positive	1	10		4				0	6		9				12	3		0				11	4		0			
Clinical stage				0.065		0.625	0.126	0.510			0.879		0.170	0.458	**0.031**			0.439		NA	0.439	NA			0.734		0.556	0.795	0.550
	I-II	11	33		11				7	31		18				51	5		0				42	13		0			
	III-IV	48	70		38				14	67		74				136	20		0				121	33		1			
Smoking				0.486		0.208	0.311	0.293			0.849		0.980	0.909	0.815			0.618		NA	0.618	NA			0.317		0.136	0.215	0.149
	No	28	58		32				11	56		51				106	12		0				89	27		2			
	Yes	36	60		26				12	56		55				108	15		0				100	22		0			
Drinking				0.381		1.000	0.533	0.559			0.522		0.434	0.458	0.651			0.497		NA	0.497	NA			0.119		0.166	0.077	0.188
	No	32	67		29				14	60		55				112	16		0				96	31		2			
	Yes	32	51		29				9	52		51				102	11		0				93	18		0			
Family history of cancer				0.797		0.191	0.759	1.000			0.279		0.918	0.560	0.061			0.486		NA	0.486	NA			0.786		0.508	0.701	0.513
	No	52	94		52				20	86		93				178	21		0				155	41		2			
	Yes	12	24		6				3	26		13				36	6		0				34	8		0			
SCC				0.909		0.731	0.819	0.737			0.455		0.466	0.448	0.861			0.073		NA	0.073	NA			0.128		0.643	0.156	0.605
	Normal	25	39		20				8	39		36				74	10		0				65	17		1			
	Increased	6	10		6				1	11		10				16	6		0				14	8		0			
CEA				0.379		0.125	0.155	0.147			0.978		0.189	0.539	**0.036**			0.897		NA	0.897	NA			0.662		0.652	0.585	0.668
	Normal	16	34		18				6	31		30				58	10		0				49	16		2			
	Increased	3	3		0				1	5		0				5	1		0				5	1		0			
CYFRA				0.901		0.782	0.849	0.803			0.200		0.393	0.233	0.824			0.082		NA	0.082	NA			**0.041**		NA	**0.041**	NA
	Normal	4	9		3				3	8		5				14	2		0				14	2		0			
	Increased	4	10		4				1	12		5				11	7		0				10	8		0			
EBV				0.539		0.400	0.877	0.183			0.814		0.862	0.829	1.000			0.635		NA	0.635	NA			1.000		NA	1.000	NA
	Negative	11	12		7				4	11		15				27	3		0				25	5		0			
	Positive	2	1		3				1	2		3				5	1		0				5	1		0			
Blood type				0.334		0.612	0.307	0.844			0.269		0.654	0.416	0.549			0.183		NA	0.183	NA			0.533		0.707	0.665	0.669
	A	10	23		10				4	18		21				35	8		0				31	12		0			
	B	9	19		7				6	12		17				34	1		0				29	5		1			
	AB	7	5		3				3	6		6				14	2		0				10	4		0			
	O	9	19		11				3	23		14				35	4		0				29	10		1			

Among the SNPs related to the risk of HNSCC, the heterozygous and dominant model of AKT1 rs1130233 were significantly related to lymph node metastasis and non-distant metastasis (P=0.034, P=0.046). The recessive model of AKT1 rs2494732 was significantly related to male sex, stage III-IV disease, and normal carcinoembryonic antigen (CEA) levels (P=0.041, P=0.031, P=0.036). The IL-1RN rs419598 wild-type genotype was significantly related to stage III-IV disease, the PIGR rs291102 wild-type genotype and dominance model were significantly related to normal levels of cytokeratin fragment 19 (CYFRA) (P=0.041).

## Discussion

In this study, we report for the first time an association of 28 polymorphisms with HNSCC risk and radiotherapy sensitivity in a population of individuals from the Liaoning Province of China. We found that carriers of the AKT1 rs1130233 TT genotype, dominance model (CT+TT vs. CC), recessive model (TT vs. CT+CC), and the AKT1 rs2494732 CC genotype had a reduced risk of HNSCC (*P*<0.05), whereas those with the PIGR rs291097 GA genotype, dominance model (GA+ AA vs. GG), and PIGR rs291102 dominance model (GA+ AA vs. GG) showed increased risk of HNSCC (*P*<0.05). In addition, we found that the IL-1RN rs419598, IL-21R rs2189521, and BCL2 rs2279115 genotypes were associated with reduced HNSCC risk, while the TNF rs1800630 genotype was associated with increased HNSCC risk. These findings provide experimental evidence to support these genes or SNPs as potential biomarkers of specific types of HNSCC.

It is estimated that infectious diseases and chronic inflammation account for approximately 25% of cancer-causing factors (16). Inflammation may act at multiple stages of disease development to disrupt tissue homeostasis, induce aberrant proliferative responses, modulate the tumor microenvironment, and compromise immune surveillance (50–52). Inflammatory cells and related signaling molecules can also be used by tumors to facilitate progression and metastasis by generating a favorable microenvironment, as well as promoting genetic instability and angiogenesis (53). Inflammatory physiological changes, such as oxidative stress, exert downstream genotoxic effects (54). When sustained over extended periods, these changes promote the emergence of cancer-initiating mutations (55). Genetic variations in inflammation-related genes potentially complement prediction of HNSCC risk. Gene polymorphisms are a common genetic variant. The most common polymorphic form is a base difference, termed a single nucleotide polymorphism (3).

AKT, the v-AKT murine thymoma viral oncogene homolog, maps to human chromosome 14q32.32 and encodes a 56-kDa protein, comprising 480 amino acids (56). AKT is an important effector of the PI3K/AKT/MTOR signaling pathway, and genetic mutations or abnormal protein expression can alter a variety of cellular processes including migration, proliferation, growth, and survival (57). AKT SNPs are reported to be associated with susceptibility to various cancer types, such as nasopharyngeal carcinoma (NPC), OSCC, non-small cell lung cancer, pancreatic ductal adenocarcinoma, and GC via effects on protein expression and transcriptional activity (12, 36, 56, 58–60). Zhang et al. reported that the AKT1 rs1130233 and rs2494732 AA genotypes were associated with a significantly increased susceptibility to NPC risk in a Chinese population (36). Another study also reported an association between the AKT1 polymorphism and cancer metastasis (58). Collectively, these observations indicate that our findings of associations existing between AKT1 SNPs and the risk of HNSCC are biologically relevant.

PIGR is a member of the immunoglobulin superfamily and transports immunoglobulin A (IgA) onto mucosal surfaces (61). PIGR has been described as a putative cancer biomarker in a few studies on various cancers, the majority of which indicate an association between low PIGR expression and more aggressive disease (61). Individuals carrying the PIGR rs291097 T allele have a higher risk of NPC in Guangdong Province, China (14). The PIGR rs291102 genotype is a missense mutation changing alanine to valine near an endoproteolytic cleavage site. This variant could alter the efficiency of PIGR to release the IgA-EBV complex and consequently increase the susceptibility of populations in endemic areas to develop NPC (13). Chen et al. reported that the risk of HNSCC may be associated with SNPs in the BCL2 promoter region (43). Some scholars consider that TNF-α SNPs (rs1800629, rs1799724, rs1800630, and rs1799964) may individually or, more likely, jointly affect individual susceptibility to HPV16-associated OSCC, particularly squamous cell carcinoma of the oropharynx (SCCOP) in never smokers (38). Our results are similar to the abovementioned findings, which suggests that inflammatory-related gene SNPs are closely related to the risk of HNSCC in different populations and different cases.

Following stratified analyses, we found that the IL-1RN rs419598 TT genotype and dominance model (CT+ CC vs. TT) were associated with reduced HNSCC risk in individuals older than 60 years of age. However, in those age 60 and younger, the AKT1 rs1130233 TT genotype and dominance model (CT+TT vs. CC), the IL-21R rs2189521 CT genotype and dominance model (CT+ CC vs. TT), and the BCL2 rs2279115 recessive model (TT vs. GT+GG) were associated with reduced HNSCC risk. In addition, in men, the AKT1 rs1130233 TT genotype and dominance model (CT+TT vs. CC) and the BCL2 rs2279115 TT genotype and recessive model (TT vs. GT+GG) were associated with reduced HNSCC risk. In women, however, the IL-21R rs2189521 CT genotype and dominance model (CT+ CC vs. TT) were associated with reduced HNSCC risk. Additionally, the PIGR rs291097 GA genotype and dominance model (GA+AA vs. GG) and the TNF rs1800630 AA genotype were associated with increased HNSCC risk in women. These genes are all inflammatory-related genes, and these results suggest that inflammatory-related gene SNPs are closely related to the risk of HNSCC patients.

From our research data, the correlation between various genotypes and the risk of HNSCC may be related to the differences in the distribution of different clinicopathological parameters. We also compared the genotype distribution of these polymorphisms in HNSCC patients with different clinicopathological parameters. We found that the heterozygous and dominant models of the AKT1 rs1130233 polymorphism were significantly related to non-distant metastasis. This phenomenon may indicate that the carrier of AKT1 rs1130233 dominance model has a low risk of cancer and is not prone to distant metastasis, which may indicate they have a long survival time. The IL-1RN rs419598 wild-type genotype was significantly related to stage III-IV disease, the PIGR rs291102 wild-type genotype was significantly related to normal levels of CYFRA, and the BCL2 rs2279115 wild-type genotype was significantly related to lymph node metastasis. These results suggest that individuals with the IL-1RN rs419598, or BCL2 rs2279115 polymorphisms showed a significant reduction in HNSCC risk progression, whereas those with the PIGR rs291102 dominance model had increased HNSCC risk. In addition, we found that different genotypes of some SNPs are significantly correlated with different clinicopathological parameters, such as IL-1B rs1143627, IL-4 rs2243250, and IL-4 rs2227284, IL-6 rs1800796, TNFRSF1A rs414570, TNF rs361525, COX-2 rs20417, whereas other SNPs showed no significant correlations with clinicopathological parameters in our data.

Recently, studies on the relationships between genetic polymorphisms and radiotherapy sensitivity have been reported. For example, gene polymorphisms of Wnt/beta-catenin may be novel prognostic factors for NPC patients treated with RT (62). The authors observed that the catenin beta 1 gene (CTNNB1) rs1880481 and rs3864004 polymorphisms, as well as the glycogen synthase kinase 3 beta gene (GSK3beta) rs3755557 polymorphism, were significantly associated with a poorer efficacy of RT in NPC patients (63). However, the relationship between SNPs in inflammation-related genes and the risk of HNSCC has not been reported. In this study, we found that HNSCC patients carrying the IL-4RA rs1801275 AA wild-type genotype were more sensitive to radiotherapy compared with other patients. We also analyzed the relationships between clinicopathological parameters and radiotherapy sensitivity. Age ≤ 60 years, non-smoker status, and normal levels of SCC were found to be associated with increased radiotherapy sensitivity of HNSCC patients. We expect that these results may help guide radiotherapy and concurrent radiotherapy and chemotherapy treatment plans. However, this was only a correlation study, and the support of basic science experiments is necessary.

In our study, the 28 inflammation-related gene polymorphisms we screened were previously reported in various cancers, and several SNPs have been reported in HNSCC (6, 13, 31, 34–36, 39, 42, 64, 65). Drobin et al reported the correlation and possible mechanism of VEGFA rs69947 with breast cancer and HNSCC radiotherapy sensitivity. The authors proposed that this SNP may affect protein expression, which would impact biological processes such as blood vessel growth, inflammatory cell infiltration, the immune response, DNA repair, oxidative stress and hypoxia (66). These changes may underlie the differences in correlation and sensitivity among patients. TNF-α is a cytokine that is secreted during the inflammatory process accompanying RTH and during cancer development. An SNP in the TNF-α promoter region can potentially affect the function or expression of this cytokine and thus modulate the risk of occurrence and intensity of OM and shortening of overall survival (30). To explore these possibilities, further studies are required using a larger sample size and additional in vitro and in vivo experimental analyses.

The present study has some limitations. First, the sample size was relatively small, especially for the HNSCC case group. Our results need further confirmation in larger populations. Second, only HNSCC risk was analyzed in this study. Analysis of prognostic parameters, such as overall survival and progression-free survival, is also warranted. Last, functional experiments are required to elucidate the underlying disease mechanism responsible for our observations.

In summary, we found that the AKT1 rs1130233 TT and dominance model (CT+TT vs. CC) genotypes, as well as the rs2494732 CC genotype, were associated with reduced risk of HNSCC. The PIGR rs291097 GA and dominance model (GA+AA vs. GG) genotypes, as well as the rs291102 dominance model (GA+AA vs. GG), were associated with increased risk of HNSCC. We also found that the IL-4RA rs1801275 AA genotype was significantly correlated with increased radiotherapy sensitivity of HNSCC patients. In addition, age ≤ 60 years, non-smoker status, and normal levels of SCC were found to be associated with increased radiotherapy sensitivity of HNSCC patients. We expect that future data from a larger population sample will support our results and be used to guide the comprehensive treatment and prognosis of HNSCC patients. Further investigation is needed to elucidate the molecular mechanisms governing our findings.

## Data Availability Statement

The data that support the findings of our study have been deposited into CNGB Sequence Archive (CNSA) of China National GeneBank DataBase (CNGBdb) with accession number CNP0001819.

## Ethics Statement

The studies involving human participants were reviewed and approved by the Human Ethics Committee of Liaoning Cancer Hospital. Written informed consent to participate in this study was provided by the participants’ legal guardian/next of kin.

## Author Contributions

YL and XL designed the study. HY was responsible for case screening. XK, LC, YS, and AM treated HNSCC patients. YZ was mainly for clinical information collection. YL and LZ processed, analysed data, and wrote the paper. All authors contributed to the article and approved the submitted version.

## Funding

This work was supported by grants from the Doctoral Science and Technology Research Startup Fund Project of Liaoning Province of China (2019-BS-275), the Science and Technology Fund Project of Liaoning Province of China (20180550318), and Key Laborotary of Tumor Radiosensitization and Normal Tissue Radioprotection of Liaoning Province (2018225102).

## Conflict of Interest

The authors declare that the research was conducted in the absence of any commercial or financial relationships that could be construed as a potential conflict of interest.

## References

[1] Fernandez-Mateos J Seijas-Tamayo R Klain JCA Borgonon MP Perez-Ruiz E Mesia R. Analysis of Autophagy Gene Polymorphisms in Spanish Patients With Head and Neck Squamous Cell Carcinoma. Sci Rep (2017) 7:6887. 10.1038/s41598-017-07270-0 28761177 PMC5537226

[2] Hashibe M Brennan P Benhamou S Castellsague X Chen C Curado MP. Alcohol Drinking in Never Users of Tobacco, Cigarette Smoking in Never Drinkers, and the Risk of Head and Neck Cancer: Pooled Analysis in the International Head and Neck Cancer Epidemiology Consortium. J Natl Cancer Inst (2007) 99:777–89. 10.1038/bdj.2007.638 17505073

[3] Piao Y Li Y Xu Q Liu JW Xing CZ Xie XD. Association of MTOR and AKT Gene Polymorphisms With Susceptibility and Survival of Gastric Cancer. PloS One (2015) 10:e0136447. 10.1371/journal.pone.0136447 26317520 PMC4552869

[4] Leibovici D Grossman HB Dinney CP Millikan RE Lerner S Wang Y. Polymorphisms in Inflammation Genes and Bladder Cancer: From Initiation to Recurrence, Progression, and Survival. J Clin Oncol (2005) 23:5746–56. 10.1200/JCO.2005.01.598 16110031

[5] Lu H Ouyang W Huang C. Inflammation, a Key Event in Cancer Development. Mol Cancer Res (2006) 4:221–33. 10.1158/1541-7786.MCR-05-0261 16603636

[6] Oh SS Chang SC Cai L Cordon-Cardo C Ding BG Greenland S. Single Nucleotide Polymorphisms of 8 Inflammation-Related Genes and Their Associations With Smoking-Related Cancers. Int J Cancer (2010) 127:2169–82. 10.1002/ijc.25214 PMC2932751 20112337

[7] St John MA Dohadwala M Luo J Wang G Lee G Shih H. Proinflammatory Mediators Upregulate Snail in Head and Neck Squamous Cell Carcinoma. Clin Cancer Res (2009) 15:6018–27. 10.1158/1078-0432.CCR-09-0011 PMC2782528 19789323

[8] Zhu Y Xu Y Wei Y Liang W Liao M Zhang L. Association of IL-1B Gene Polymorphisms With Nasopharyngeal Carcinoma in a Chinese Population. Clin Oncol (R Coll Radiol) (2008) 20:207–11. 10.1016/j.clon.2008.01.003 18289837

[9] Kietthubthew S Wickliffe J Sriplung H Ishida T Chonmaitree T Au WW. Association of Polymorphisms in Proinflammatory Cytokine Genes With the Development of Oral Cancer in Southern Thailand. Int J Hyg Environ Health (2010) 213:146–52. 10.1016/j.ijheh.2010.01.002 20133197

[10] Tamandani DM Sobti RC Shekari M Kaur S Huria A. Impact of Polymorphism in IL-1RA Gene on the Risk of Cervical Cancer. Arch Gynecol Obstet (2008) 277:527–33. 10.1007/s00404-007-0504-4 18008080

[11] Deans C Rose-Zerilli M Wigmore S Ross J Howell M Jackson A. Host Cytokine Genotype is Related to Adverse Prognosis and Systemic Inflammation in Gastro-Oesophageal Cancer. Ann Surg Oncol (2007) 14:329–39. 10.1245/s10434-006-9122-9 17103073

[12] Wang Y Lin L Xu H Li T Zhou Y Dan H. Genetic Variants in AKT1 Gene Were Associated With Risk and Survival of OSCC in Chinese Han Population. J Oral Pathol Med (2015) 44:45–50. 10.1111/jop.12211 25060489

[13] Hirunsatit R Kongruttanachok N Shotelersuk K Supiyaphun P Voravud N Sakuntabhai A. Polymeric Immunoglobulin Receptor Polymorphisms and Risk of Nasopharyngeal Cancer. BMC Genet (2003) 4:3. 10.1186/1471-2156-4-3 12546713 PMC149362

[14] Fan Q Jia WH Zhang RH Yu XJ Chen LZ Feng QS. Correlation of Polymeric Immunoglobulin Receptor Gene Polymorphisms to Susceptibility of Nasopharyngeal Carcinoma. Ai Zheng (2005) 24:915–8. 16086865

[15] Fu J Li Z Li N. The Association Between COX-2 Gene Rs5275 Polymorphism and Nasopharyngeal Carcinoma Risk. Pathol Res Pract (2018) 214:1579–82. 10.1016/j.prp.2018.07.028 30087034

[16] Murata M. Inflammation and Cancer. Environ Health Prev Med (2018) 23:50. 10.1186/s12199-018-0740-1 30340457 PMC6195709

[17] Huang SH O’Sullivan B. Overview of the 8th Edition TNM Classification for Head and Neck Cancer. Curr Treat Options Oncol (2017) 18:40. 10.1007/s11864-017-0484-y 28555375

[18] Powrozek T Mlak R Brzozowska A Mazurek M Golebiowski P Malecka-Massalska T. Relationship Between TNF-Alpha -1031T/C Gene Polymorphism, Plasma Level of TNF-Alpha, and Risk of Cachexia in Head and Neck Cancer Patients. J Cancer Res Clin Oncol (2018) 144:1423–34. 10.1007/s00432-018-2679-4 PMC6061036 29802455

[19] Cui XB Wang DD Zhang HY Li TT Jin TT Peng H. Tumor Necrosis Factor-Alpha Gene 308G/a Polymorphism is Not Associated With Esophageal Squamous Cell Carcinoma Risk in Kazakh Patients. Int J Clin Exp Pathol (2015) 8:9293–9. PMC4583911 26464679

[20] Singh PK Bogra J Chandra G Ahmad MK Gupta R Kumar V. Association of TNF-Alpha (-238 and -308) Promoter Polymorphisms With Susceptibility of Oral Squamous Cell Carcinoma in North Indian Population. Cancer Biomark (2015) 15:125–31. 10.3233/CBM-140444 PMC12928520 25519014

[21] Hsu HJ Yang YH Shieh TY Chen CH Kao YH Yang CF. Role of Cytokine Gene (Interferon-Gamma, Transforming Growth Factor-Beta1, Tumor Necrosis Factor-Alpha, Interleukin-6, and Interleukin-10) Polymorphisms in the Risk of Oral Precancerous Lesions in Taiwanese. Kaohsiung J Med Sci (2014) 30:551–8. 10.1016/j.kjms.2014.09.003 PMC11916204 25458044

[22] Zhang C Sturgis EM Zheng H Zafereo ME Wei Q Li G. TNF-Alpha Promoter Polymorphisms and Risk of Recurrence in Patients With Squamous Cell Carcinomas of the Nonoropharynx. Int J Cancer (2014) 135:1615–24. 10.1002/ijc.28793 PMC4107187 24550071

[23] Mendes SO dos Santos M Peterle GT Maia Lde L Stur E Agostini LP. HIF-1alpha Expression Profile in Intratumoral and Peritumoral Inflammatory Cells as a Prognostic Marker for Squamous Cell Carcinoma of the Oral Cavity. PloS One (2014) 9:e84923. 10.1371/journal.pone.0084923 24416312 PMC3887011

[24] Al-toub M Almusa A Almajed M Al-Nbaheen M Kassem M Aldahmash A. Pleiotropic Effects of Cancer Cells’ Secreted Factors on Human Stromal (Mesenchymal) Stem Cells. Stem Cell Res Ther (2013) 4:114. 10.1186/scrt325 24405819 PMC3854757

[25] de Jesus GP Ribeiro FA de Moura CF Gollucke AP Oshima CT Ribeiro DA. Anti-Tumor Activity of Grape Juice Concentrate in the Rat Tongue Two-Stage Initiation-Promotion Protocol Induced by 4-Nitroquinoline 1-Oxide. Toxicol Mech Methods (2014) 24:276–83. 10.3109/15376516.2014.881944 24401099

[26] Erdogan M Karadeniz M Ozbek M Ozgen AG Berdeli A. Interleukin-10 Gene Polymorphism in Patients With Papillary Thyroid Cancer in Turkish Population. J Endocrinol Invest (2008) 31:750–4. 10.1007/BF03349252 18997484

[27] Chiang SL Chen PH Lee CH Ko AM Lee KW Lin YC. Up-Regulation of Inflammatory Signalings by Areca Nut Extract and Role of Cyclooxygenase-2 -1195G>a Polymorphism Reveal Risk of Oral Cancer. Cancer Res (2008) 68:8489–98. 10.1158/0008-5472.CAN-08-0823 18922923

[28] de Luis DA Sagrado MG Vallejo LA Carcedo LM Izaola O Cuellar L. Influence of G308A Polymorphism of Tumor Necrosis Factor-Alpha Gene on Inflammatory Markers in Postsurgical Head and Neck Cancer Patients With Early Enteral Nutrition. Nutrition (2007) 23:529–32. 10.1016/j.nut.2007.04.011 17560079

[29] Pu X Wang L Chang JY Hildebrandt MA Ye Y Lu C. Inflammation-Related Genetic Variants Predict Toxicity Following Definitive Radiotherapy for Lung Cancer. Clin Pharmacol Ther (2014) 96:609–15. 10.1038/clpt.2014.154 PMC4206576 25054431

[30] Mlak R Powrozek T Brzozowska A Homa-Mlak I Mazurek M Golebiowski P. The Relationship Between TNF-Alpha Gene Promoter Polymorphism (- 1211 T > C), the Plasma Concentration of TNF-Alpha, and Risk of Oral Mucositis and Shortening of Overall Survival in Patients Subjected to Intensity-Modulated Radiation Therapy Due to Head and Neck Cancer. Support Care Cancer (2019) 28(2):531–40. 10.1007/s00520-019-04838-6 PMC6954128 31076897

[31] Wu MY Huang SJ Yang F Qin XT Liu D Ding Y. Detection of Nasopharyngeal Carcinoma Susceptibility With Single Nucleotide Polymorphism Analysis Using Next-Generation Sequencing Technology. Oncotarget (2017) 8:52708–23. 10.18632/oncotarget.17085 PMC5581063 28881764

[32] Brzozowska A Powrozek T Homa-Mlak I Mlak R Ciesielka M Golebiowski P. Polymorphism of Promoter Region of TNFRSF1A Gene (-610 T > G) as a Novel Predictive Factor for Radiotherapy Induced Oral Mucositis in HNC Patients. Pathol Oncol Res (2018) 24:135–43. 10.1007/s12253-017-0227-1 PMC5736772 28401452

[33] Boaventura P Duraes C Mendes A Costa NR Chora I Ferreira S. IL6-174 G>C Polymorphism (Rs1800795) Association With Late Effects of Low Dose Radiation Exposure in the Portuguese Tinea Capitis Cohort. PloS One (2016) 11:e0163474. 10.1371/journal.pone.0163474 27662210 PMC5035001

[34] Sousa H Bastos MJ Ribeiro J Oliveira S Breda E Catarino R. 5’UTR +24T>C CR2 is Not Associated With Nasopharyngeal Carcinoma Development in the North Region of Portugal. Oral Dis (2016) 22:280–4. 10.1111/odi.12436 26748973

[35] Sousa H Mesquita L Ribeiro J Catarino R Breda E Medeiros R. Polymorphisms in Host Immune Response Associated Genes and Risk of Nasopharyngeal Carcinoma Development in Portugal. Immunobiology (2016) 221:145–52. 10.1016/j.imbio.2015.09.015 26391153

[36] Zhang X Chen X Zhai Y Cui Y Cao P Zhang H. Combined Effects of Genetic Variants of the PTEN, AKT1, MDM2 and P53 Genes on the Risk of Nasopharyngeal Carcinoma. PloS One (2014) 9:e92135. 10.1371/journal.pone.0092135 24632578 PMC3954877

[37] Pu X Hildebrandt MA Lu C Roth JA Stewart DJ Zhao Y. Inflammation-Related Genetic Variations and Survival in Patients With Advanced non-Small Cell Lung Cancer Receiving First-Line Chemotherapy. Clin Pharmacol Ther (2014) 96:360–9. 10.1038/clpt.2014.89 PMC4141040 24755914

[38] Jin L Sturgis EM Zhang Y Huang Z Song X Li C. Association of Tumor Necrosis Factor-Alpha Promoter Variants With Risk of HPV-Associated Oral Squamous Cell Carcinoma. Mol Cancer (2013) 12:80. 10.1186/1476-4598-12-80 23870134 PMC3725173

[39] Fan Q He JF Wang QR Cai HB Sun XG Zhou XX. Functional Polymorphism in the 5’-UTR of CR2 is Associated With Susceptibility to Nasopharyngeal Carcinoma. Oncol Rep (2013) 30:11–6. 10.3892/or.2013.2421 PMC3729234 23612877

[40] Gaur P Mittal M Mohanti B Das S. Functional Variants of IL4 and IL6 Genes and Risk of Tobacco-Related Oral Carcinoma in High-Risk Asian Indians. Oral Dis (2011) 17:720–6. 10.1111/j.1601-0825.2011.01831.x 21771210

[41] Mittal M Kapoor V Mohanti BK Das SN. Functional Variants of COX-2 and Risk of Tobacco-Related Oral Squamous Cell Carcinoma in High-Risk Asian Indians. Oral Oncol (2010) 46:622–6. 10.1016/j.oraloncology.2010.06.002 20620096

[42] Farhat K Hassen E Gabbouj S Bouaouina N Chouchane L. Interleukin-10 and Interferon-Gamma Gene Polymorphisms in Patients With Nasopharyngeal Carcinoma. Int J Immunogenet (2008) 35:197–205. 10.1111/j.1744-313X.2008.00752.x 18312596

[43] Chen K Hu Z Wang LE Sturgis EM El-Naggar AK Zhang W. Single-Nucleotide Polymorphisms At the TP53-Binding or Responsive Promoter Regions of BAX and BCL2 Genes and Risk of Squamous Cell Carcinoma of the Head and Neck. Carcinogenesis (2007) 28:2008–12. 10.1093/carcin/bgm172 17693666

[44] Guo W Wang N Wang YM Li Y Wen DG Chen ZF. Interleukin-10 -1082 Promoter Polymorphism is Not Associated With Susceptibility to Esophageal Squamous Cell Carcinoma and Gastric Cardiac Adenocarcinoma in a Population of High-Incidence Region of North China. World J Gastroenterol (2005) 11:858–62. 10.3748/wjg.v11.i6.858 PMC4250597 15682481

[45] Xu Q Yuan Y Sun LP Gong YH Xu Y Yu XW. Risk of Gastric Cancer is Associated With the MUC1 568 a/G Polymorphism. Int J Oncol (2009) 35:1313–20. 10.3892/ijo_00000449 19885554

[46] Bonaparte E Pesenti C Fontana L Falcone R Paganini L Marzorati A. Molecular Profiling of Lung Cancer Specimens and Liquid Biopsies Using MALDI-TOF Mass Spectrometry. Diagn Pathol (2018) 13:4. 10.1186/s13000-017-0683-7 29368620 PMC6389067

[47] Yang XD Zhao SF Zhang Q Li W Wang YX Hong XW. Gelsolin Rs1078305 and Rs10818524 Polymorphisms Were Associated With Risk of Oral Squamous Cell Carcinoma in a Chinese Han Population. Biomarkers (2016) 21:267–71. 10.3109/1354750X.2015.1134664 26848502

[48] Singhal N Kumar M Kanaujia PK Virdi JS. MALDI-TOF Mass Spectrometry: an Emerging Technology for Microbial Identification and Diagnosis. Front Microbiol (2015) 6:791. 10.3389/fmicb.2015.00791 26300860 PMC4525378

[49] Eisenhauer EA Therasse P Bogaerts J Schwartz LH Sargent D Ford R. New Response Evaluation Criteria in Solid Tumours: Revised RECIST Guideline (Version 1.1). Eur J Cancer (2009) 45:228–47. 10.1016/j.ejca.2008.10.026 19097774

[50] Hanahan D Weinberg RA. Hallmarks of Cancer: the Next Generation. Cell (2011) 144:646–74. 10.1016/j.cell.2011.02.013 21376230

[51] Elinav E Nowarski R Thaiss CA Hu B Jin C Flavell RA. Inflammation-Induced Cancer: Crosstalk Between Tumours, Immune Cells and Microorganisms. Nat Rev Cancer (2013) 13:759–71. 10.1038/nrc3611 24154716

[52] Jinushi M. Yin and Yang of Tumor Inflammation: How Innate Immune Suppressors Shape the Tumor Microenvironments. Int J Cancer (2014) 135:1277–85. 10.1002/ijc.28626 24272248

[53] Coussens LM Werb Z. Inflammation and Cancer. Nature (2002) 420:860–7. 10.1038/nature01322 PMC2803035 12490959

[54] Atsumi T Singh R Sabharwal L Bando H Meng J Arima Y. Inflammation Amplifier, a New Paradigm in Cancer Biology. Cancer Res (2014) 74:8–14. 10.1158/0008-5472.CAN-13-2322 24362915

[55] Buas MF He Q Johnson LG Onstad L Levine DM Thrift AP. Germline Variation in Inflammation-Related Pathways and Risk of Barrett’s Oesophagus and Oesophageal Adenocarcinoma. Gut (2017) 66:1739–47. 10.1136/gutjnl-2016-311622 PMC5296402 27486097

[56] Kim MJ Kang HG Lee SY Jeon HS Lee WK Park JY. AKT1 Polymorphisms and Survival of Early Stage non-Small Cell Lung Cancer. J Surg Oncol (2012) 105:167–74. 10.1002/jso.22071 21842521

[57] De Marco C Rinaldo N Bruni P Malzoni C Zullo F Fabiani F. Multiple Genetic Alterations Within the PI3K Pathway are Responsible for AKT Activation in Patients With Ovarian Carcinoma. PloS One (2013) 8:e55362. 10.1371/journal.pone.0055362 23408974 PMC3567053

[58] Li Q Yang J Yu Q Wu H Liu B Xiong H. Associations Between Single-Nucleotide Polymorphisms in the PI3K-PTEN-AKT-Mtor Pathway and Increased Risk of Brain Metastasis in Patients With non-Small Cell Lung Cancer. Clin Cancer Res (2013) 19:6252–60. 10.1158/1078-0432.CCR-13-1093 24077347

[59] Avan A Avan A Le Large TY Mambrini A Funel N Maftouh M. AKT1 and SELP Polymorphisms Predict the Risk of Developing Cachexia in Pancreatic Cancer Patients. PloS One (2014) 9:e108057. 10.1371/journal.pone.0108057 25238546 PMC4169595

[60] Wang X Lin Y Lan F Yu Y Ouyang X Wang X. A GG Allele of 3’-Side AKT1 SNP is Associated With Decreased AKT1 Activation and Better Prognosis of Gastric Cancer. J Cancer Res Clin Oncol (2014) 140:1399–411. 10.1007/s00432-014-1663-x PMC11824104 24737346

[61] Fristedt R Gaber A Hedner C Nodin B Uhlen M Eberhard J. Expression and Prognostic Significance of the Polymeric Immunoglobulin Receptor in Esophageal and Gastric Adenocarcinoma. J Transl Med (2014) 12:83. 10.1186/1479-5876-12-83 24694107 PMC4021601

[62] Ban S Konomi C Iwakawa M Yamada S Ohno T Tsuji H. Radiosensitivity of Peripheral Blood Lymphocytes Obtained From Patients With Cancers of the Breast, Head and Neck or Cervix as Determined With a Micronucleus Assay. J Radiat Res (2004) 45:535–41. 10.1269/jrr.45.535 15635263

[63] Yu J Huang Y Liu L Wang J Yin J Huang L. Genetic Polymorphisms of Wnt/Beta-Catenin Pathway Genes are Associated With the Efficacy and Toxicities of Radiotherapy in Patients With Nasopharyngeal Carcinoma. Oncotarget (2016) 7:82528–37. 10.18632/oncotarget.12754 PMC5347711 27769064

[64] Caetano MS Zhang H Cumpian AM Gong L Unver N Ostrin EJ. IL6 Blockade Reprograms the Lung Tumor Microenvironment to Limit the Development and Progression of K-Ras-Mutant Lung Cancer. Cancer Res (2016) 76:3189–99. 10.1158/0008-5472.CAN-15-2840 PMC4891282 27197187

[65] Pasvenskaite A Vilkeviciute A Liutkeviciene R Gedvilaite G Liutkevicius V Uloza V. Associations of IL6 Rs1800795, BLK Rs13277113, TIMP3 Rs9621532, IL1RL1 Rs1041973 and IL1RAP Rs4624606 Single Gene Polymorphisms With Laryngeal Squamous Cell Carcinoma. Gene (2020) 747:144700. 10.1016/j.gene.2020.144700 32330537

[66] Drobin K Marczyk M Halle M Danielsson D Papiez A Sangsuwan T. Molecular Profiling for Predictors of Radiosensitivity in Patients With Breast or Head-and-Neck Cancer. Cancers (Basel) (2020) 12(3):753–71. 10.3390/cancers12030753 PMC7140105 32235817

